# A FOXO1-dependent transcription network is a targetable vulnerability of mantle cell lymphomas

**DOI:** 10.1172/JCI160767

**Published:** 2022-12-15

**Authors:** Ja-Young Jang, Inah Hwang, Heng Pan, Jun Yao, Lapo Alinari, Eddie Imada, Claudio Zanettini, Michael J. Kluk, Yizhe Wang, Yunkyoung Lee, Hua V. Lin, Xiangao Huang, Maurizio Di Liberto, Zhengming Chen, Karla V. Ballman, Lewis C. Cantley, Luigi Marchionni, Giorgio Inghirami, Olivier Elemento, Robert A. Baiocchi, Selina Chen-Kiang, Sandro Belvedere, Hongwu Zheng, Jihye Paik

**Affiliations:** 1 Department of Pathology and Laboratory Medicine and; 2 Caryl and Israel Englander Institute for Precision Medicine, Department of Physiology and Biophysics, Weill Cornell Medicine, New York, New York, USA.; 3 Department of Molecular and Cellular Oncology, University of Texas MD Anderson Cancer Center, Houston, Texas, USA.; 4 Division of Hematology, Department of Internal Medicine, College of Medicine, The Ohio State University, Columbus, Ohio, USA.; 5 Sandra and Edward Meyer Cancer Center, Weill Medical College of Cornell University, New York, New York, USA.; 6 Forkhead BioTherapeutics Inc., New York, New York, USA.; 7 Division of Biostatistics, Department of Population Health Sciences, and; 8 Department of Medicine, Weill Cornell Medicine, New York, New York, USA.

**Keywords:** Oncology, Lymphomas

## Abstract

Targeting lineage-defined transcriptional dependencies has emerged as an effective therapeutic strategy in cancer treatment. Through screening for molecular vulnerabilities of mantle cell lymphoma (MCL), we identified a set of transcription factors (TFs) including FOXO1, EBF1, PAX5, and IRF4 that are essential for MCL propagation. Integrated chromatin immunoprecipitation and sequencing (ChIP-Seq) with transcriptional network reconstruction analysis revealed FOXO1 as a master regulator that acts upstream in the regulatory TF hierarchy. FOXO1 is both necessary and sufficient to drive MCL lineage commitment through supporting the lineage-specific transcription programs. We further show that FOXO1, but not its close paralog FOXO3, can reprogram myeloid leukemia cells and induce B-lineage gene expression. Finally, we demonstrate that cpd10, a small molecule identified from an enriched FOXO1 inhibitor library, induces a robust cytotoxic response in MCL cells in vitro and suppresses MCL progression in vivo. Our findings establish FOXO1 inhibition as a therapeutic strategy targeting lineage-driven transcriptional addiction in MCL.

## Graphical abstract

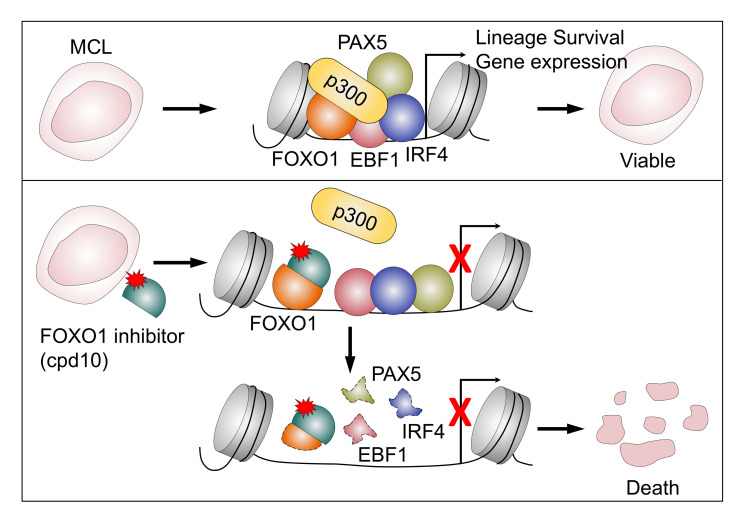

## Introduction

Mantle cell lymphoma (MCL) is a lethal mature B cell lymphoma manifested by cyclin D1 overexpression due to a t(11;14)(q13;q32) chromosomal translocation and mutations of genes associated with cell proliferation and survival, including *CDKN2A*, *ATM*, or *TP53* (1). Over the years, a large variety of conventional and targeted therapeutic strategies, including combination immunochemotherapy and autologous stem cell transplantation, have been used clinically to manage this disease (2–5). More recently, efforts in precision oncology have led to the advancement of new therapeutics for the treatment of refractory and resistant MCL, such as anti-CD19 chimeric antigen receptor (CAR) T cell therapy, anti-BCL2, or non-covalent Bruton’s tyrosine kinase (BTK) inhibitors (6–11). But despite initial success, therapy resistance nonetheless emerges (12). Thus, there is a need to identify new therapeutic targets and treatment strategies for MCL patients.

Oncogenic events occur in the context of cellular identity that is established and sustained by lineage-specific transcriptional programs (13, 14). Suppression of the core lineage transcription factors (TFs) within those programs dissolves pro-oncogenic gene expression networks and induces cancer cell death. Targeting the TF-defined lineage dependencies has thus emerged as an important therapeutic avenue in modern cancer treatment, as highlighted by the enduring clinical success of agents that target the estrogen receptor for breast cancers (15, 16) or the androgen receptor for prostate cancers (17, 18). Therefore, elucidation of MCL lineage-survival transcriptional programs may also help to unravel a new class of therapeutic targets.

FOXO TFs play an evolutionarily conserved role in many biological processes, including cellular metabolism, stress response, and tumorigenesis (19). The PI3K/AKT/FOXO signaling cascade is known to regulate cellular growth and proliferation. Inactivation of FOXO1 and its closely related paralogs FOXO3 and FOXO4 has been shown to promote tumorigenesis in the mesodermal lineages (20), kidney (21), and prostate (22) in animal models. In addition to its well-known tumor suppressor effects, FOXO1 has been linked to the process of mature B cell development and germinal center dark zone creation (23–28). In particular, a significant fraction of B cell–originated lymphomas, including approximately 12% of Burkitt lymphomas (BLs) and approximately 9% of diffuse large B cell lymphomas (DLBCLs), carry activating FOXO1 missense mutations (29, 30), suggesting that FOXO1 can act as a tumor suppressor or a lineage-survival oncogene in a strictly context-dependent manner.

Despite the broad therapeutic promise of targeting lineage-survival transcriptional programs, a major hurdle has been the identification of pharmacologically effective compounds. In fact, outside nuclear receptors, TFs have been considered undruggable owing to their intrinsically disordered structures and general lack of defined small-molecule binding pockets (31–33). In tackling this problem, we combined unbiased genetic and pharmacological approaches to catalog the MCL transcriptional lineage-survival programs. Our CRISPR/Cas9–based TF library screening identified EBF1, FOXO1, IRF4, and PAX5 as the top lineage-defined TFs necessary for MCL survival and proliferation. Integrated chromatin immunoprecipitation and sequencing (ChIP-Seq) and transcriptional network reconstruction analysis revealed FOXO1 at the upstream regulatory TF hierarchy that drives the MCL lineage-survival program. Through a small-molecule library screen, we further identified a FOXO1-targeted lead compound. Finally, we demonstrate that genetic or pharmacological targeting of FOXO1 in MCL cells could induce a robust cytotoxic response. These findings establish FOXO1 as a lineage-specific survival factor that primes MCL lineage transcriptional regulation to promote disease progression and thus represents a bona fide therapeutic target for MCL.

## Results

### Domain-focused CRISPR/Cas9 screening identifies core TFs essential for MCL proliferation and survival.

**Figure 1:**
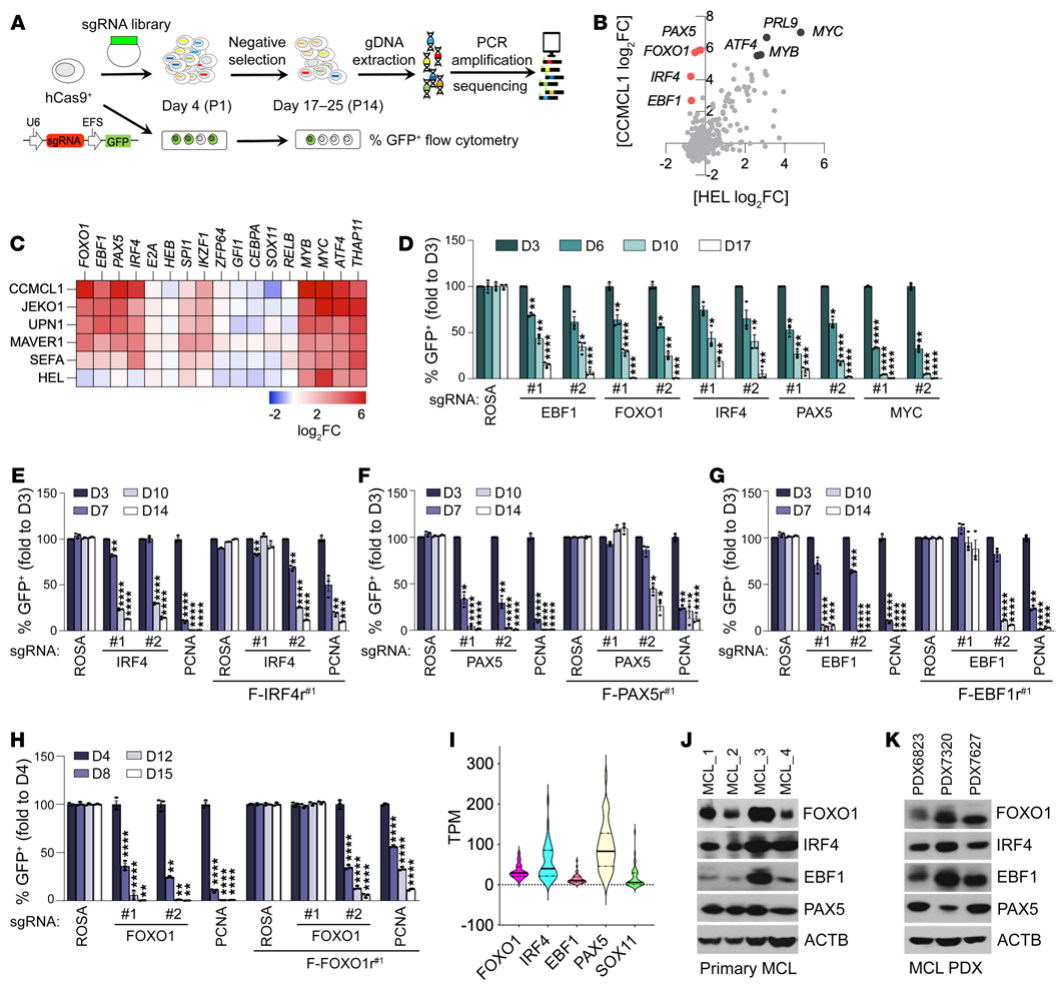
Domain-focused CRISPR/Cas9 screening identifies core TFs essential for MCL proliferation and survival. (**A**) Experimental schematic for the CRISPR/Cas9 screen and the competition-based GFP dropout proliferation assay. (**B**) Scatterplot analysis of TF dependencies in CCMCL1 cells (*y* axis) versus HEL cells (*x* axis) ranked by the average sgRNA log_2_ fold change (log_2_FC) of each gene in the pooled CRISPR screen. (**C**) Heatmap depicts log_2_FC of sgRNA abundance of selected genes (averaging each independent sgRNA targeting a gene). (**D**) Competition-based proliferation assays to validate the results from the pooled screen. Experiments were conducted by transduction of Cas9-expressing JEKO1 cells with indicated lentivirus sgRNAs that coexpress a GFP reporter. Plotted is the percentage of GFP-positive cells (normalized to the day 3 measurement) at the indicated time points during culturing. sgRNAs targeting ROSA and MYC are included as a nontargeting negative control and a positive control, respectively. (**E**–**H**) Verification of on-target effects of sgRNAs against IRF4 (**E**), PAX5 (**F**), EBF1 (**G**), and FOXO1 (**H**). Competition-based proliferation assays in CCMCL1 cells expressing control or 3× FLAG–tagged and CRISPR-resistant synonymous IRF4 (F-IRF4r^#1^), PAX5 (F-PAX5r^#1^), EBF1 (F-EBF1r^#1^), and FOXO1 (F-FOXO1r^#1^) mutants. The indicated CRISPR-resistant synonymous mutants were designed specifically for sgIRF4#1, sgPAX5#1, sgEBF1#1, and sgFOXO1#1. (**I**) Violin plots of RNA expression levels in transcripts per million (TPM) of indicated TFs in patient MCL cells (*n* = 37). RNA-Seq data were reanalyzed from GSE141336 of Zhao et al. (38). (**J** and **K**) Immunoblot analysis of patient MCL cells (**J**) and patient-derived xenografts (PDX) (**K**). (**D**–**H**) Data represent mean ± SEM (*n* = 3). Results are representative of 2 independent experiments. Statistical analysis was performed using 1-way ANOVA with Tukey’s multiple-comparison test. *P < 0.05, **P < 0.001, ***P < 0.0005, ****P < 0.0001.

To characterize MCL lineage dependencies, we conducted a CRISPR/Cas9–based negative selection screening using a domain-focused single-guide RNA (sgRNA) library consisting of 8,908 sgRNAs targeting 1,427 human TFs (Figure 1A) (34). Five MCL lines (CCMCL1, JEKO1, UPN1, MAVER1, and SEFA) plus an acute myeloid leukemia (AML) line (HEL, non-MCL control) were first transduced with lenti-Cas9 before the introduction of the pooled sgRNA library. After 14 population doublings, the relative impact of each sgRNA on cell growth was assessed via DNA sequencing–based quantification of sgRNA abundance. Spike-in positive and negative control sgRNAs included in the library validated the overall accuracy of the screening strategy (Supplemental Figure 1A; supplemental material available online with this article; https://doi.org/10.1172/JCI160767DS1). As expected, we found that most TF dependencies were nonselective and present in all 6 cell lines, such as ATF4, MYB, MYC, SPI1, and THAP11 (Figure 1, B and C, Supplemental Figure 1B, and Supplemental Table 1). To catalog the selective TF dependencies for MCL cells, we ranked each TF based on its relative essentiality in the 5 MCL cell lines versus the control AML cell line HEL. Of the total 1,427 TFs, we identified 4 TFs — EBF1, FOXO1, IRF4, and PAX5 — as the top MCL-selective hits whose sgRNA-mediated targeting had a minimal impact on HEL cell proliferation but caused a severe growth arrest phenotype in all 5 MCL lines (Figure 1, B and C, and Supplemental Figure 1B). Cross-checking with the published screen data of the same sgRNA library in 33 cancer cell lines of various tissue origins (34) revealed that the 4 identified TFs were not essential for the survival of leukemia, sarcoma, lung cancer, or pancreatic cancer cells, suggesting that they are lineage-specific survival TFs of MCL cells. Furthermore, strongly biased dependency scores of 4 TFs extracted from Project Achilles (DepMap 22Q2; ref. 35) from 1,086 cell lines encompassing 27 lineage subtypes confirmed their selective essentiality in the subset of 42 cell lines of B lymphocyte origin and diseases (i.e., non-Hodgkin’s lymphoma) (Supplemental Figure 1C).

To validate the findings of our pooled screens, we conducted competition-based proliferation assays of individual sgRNAs against these 4 identified candidates (Figure 1A). Consistent with our pooled CRISPR screening results, transduction of the green fluorescent protein (GFP) reporter coexpressing sgRNA constructs targeting EBF1, FOXO1, IRF4, or PAX5 severely impaired the growth of MCL cell lines JEKO1 (Figure 1D) and CCMCL1 (Supplemental Figure 1D). The on-target effects of the individual sgRNAs were confirmed by complementation experiments in which pre-transduction of CCMCL1 cells with a CRISPR-resistant cDNA of *EBF1*, *FOXO1*, *IRF4*, or *PAX5* rescued the growth arrest phenotype caused by the respective sgRNAs targeting those TFs (Figure 1, E–H, and Supplemental Figure 1, E and F). By contrast, sgRNAs targeting 3 reported MCL oncogenic driver TFs (SOX11, RELA, and RELB) (36, 37) caused only a modest dropout in CCMCL1, MAVER1, and JEKO1 cells (Supplemental Figure 2, A–D), suggesting that these are not commonly essential MCL lineage-survival TFs. Reanalysis of publicly accessible data sets (38) confirmed that transcripts of EBF1, FOXO1, IRF4, and PAX5 are expressed across MCL patients, with PAX5 > IRF4 > FOXO1 > EBF1 as the median expression order (Figure 1I). The protein expression was further confirmed in primary MCL cells and patient-derived xenograft samples (Figure 1, J and K, and Supplemental Table 2).

EBF1, FOXO1, IRF4, and PAX5 have previously been implicated in B cell development (39). To test whether they were broadly essential, we performed a competition-based assay in cells of B cell–origin malignancies such as the DLBCL cell line OCI-Ly1 (Supplemental Figure 2E) and the BL cell lines BJAB (Supplemental Figure 2F) and DG75 (Supplemental Figure 2G). Notably, while PAX5 was essential for DG75 cells, depletion of FOXO1, EBF1, or IRF4 had only minor or modest effects on the growth of DG75 and OCI-Ly1 cells and no effect on BJAB cells, indicating that these TFs are not universally required for B cell malignancies. In addition, we found that the 4 TFs were totally dispensable for the non–B cell malignancies, including the AML cell line HEL (Supplemental Figure 2H), the osteosarcoma cell line U2OS (Supplemental Figure 2I), the non–small cell lung carcinoma cell line H1299 (Supplemental Figure 2J), and the cervical cancer cell line HeLa (Supplemental Figure 2K). As a positive control for CRISPR competency, targeting of proliferating cell nuclear antigen (PCNA) by sgPCNA reduced the viability of the individual cancer cell lines included in this study (Supplemental Figure 2L), ruling out the possibility of incomparable genome editing efficiency. Together, our CRISPR screening identified EBF1, FOXO1, IRF4, and PAX5 as MCL lineage–specific survival TFs.

### Colocalization of MCL survival TFs facilitates collaborative regulation of B cell fate genes.

**Figure 2:**
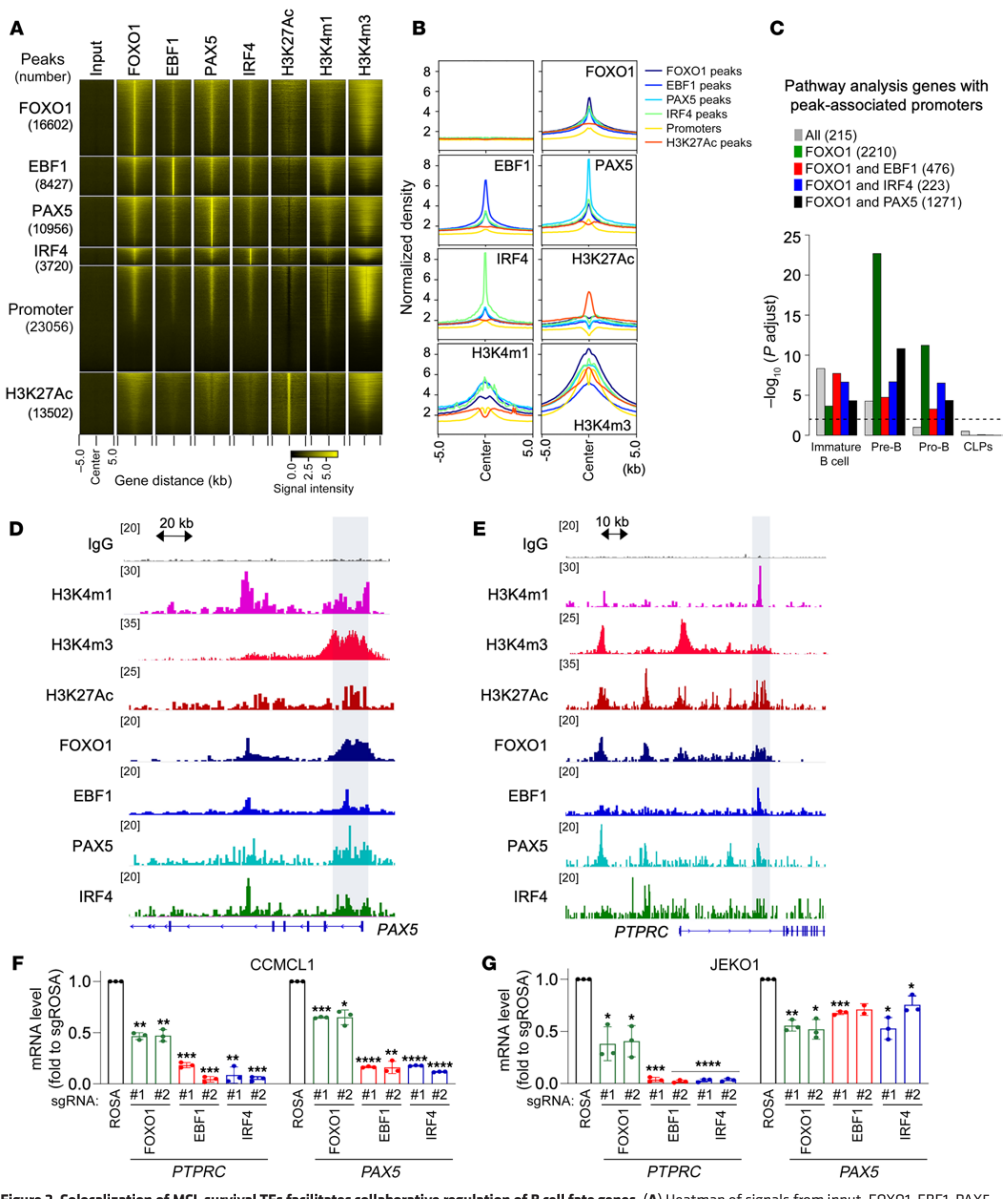
Colocalization of MCL survival TFs facilitates collaborative regulation of B cell fate genes. (**A**) Heatmap of signals from input, FOXO1, EBF1, PAX5, IRF4, H3K27ac, H3K4m1, and H3K4m3 ChIP-Seq from CCMCL1 cells at ChIP-Seq peaks (number) as well as promoters of UCSC genes. The window extends 5 kb in each direction from the center of ChIP-Seq peaks or transcription start sites. (**B**) Histogram view of **A**. (**C**) Enrichment analysis of genes (number) with ChIP-Seq peak–associated promoters within gene sets highly expressed at each of the 4 developmental stages in the healthy B cells. Adjusted *P* values were calculated by a hypergeometric test followed by a Benjamini-Hochberg procedure. The black dashed line represents FDR cutoff 0.01. CLP, common lymphoid progenitor. (**D** and **E**) Visualization of representative ChIP-Seq tracks for indicated B cell genes. (**F** and **G**) Verification of TF regulation in MCL. At 72 hours after transduction of indicated sgRNAs, CCMCL1 or JEKO1 cells were analyzed for *PTPRC* and *PAX5* mRNA levels by RT-qPCR). Data represent mean ± SEM (*n* = 3). Results are representative of 3 or 4 independent experiments. Statistical analysis in **F** and **G** was performed using 2-tailed unpaired Student’s *t* test. *P < 0.05, **P < 0.005, ***P = 0.0005, ****P < 0.0001.

Previous studies reported FOXO1, EBF1, PAX5, and IRF4 as TFs involved in B cell development and B-lineage commitment (39, 40). To understand the transcription programs that are regulated by these TFs in MCL cells, we performed ChIP-Seq to define their genome-wide occupancy. Analysis of the ChIP-Seq data found a colocalization of the 4 TFs at chromatin regions of CCMCL1 cells (Figure 2, A and B). The specificity of EBF1, PAX5, and IRF4 chromatin colocalization with FOXO1 was further confirmed by comparison of their peak enrichment with randomly shuffled regions (Supplemental Figure 3, A and B). When ChIP-Seq peaks were aligned, we found the colocalization of the 4 TFs at a set of target genes. Specifically, we found that FOXO1 had the largest number of binding sites that were associated with 5,261 genes. Among those genes, 24.6% were commonly occupied by EBF1, 42.4% by PAX5, 15.6% by IRF4 (Supplemental Figure 3C), and 4.1% (215 genes; Supplemental Table 3) by all 4 TFs. Notably, the 4 TF-colocalized genomic regions were also enriched for H3K27ac, H3K4m1, or H3K4m3 marks, indicating active promoter/enhancer regions (Figure 2B). To determine the relevance of their colocalization to B-lineage development, we next performed UpSet plot analysis to identify the commonly associated genes among the 4 TFs (Supplemental Figure 3C). The biggest overlap was discovered between FOXO1-occupied genes and those occupied by EBF1 (66.4%), PAX5 (66.1%), or IRF4 (75.6%) out of 15 comparison groups, indicating that FOXO1 may operate as a significant regulatory TF. Based on the finding, we performed enrichment analysis of genes occupied by FOXO1 together with another TF using a previously described stage-specific B cell transcription signature (41). As predicted, we found an enrichment of genes co-occupied by FOXO1 and other TFs at immature B cell, pre–B cell, and pro–B cell genes but not at genes associated with common lymphoid progenitors (FDR < 0.01; Figure 2C). This finding suggests that FOXO1, along with EBF1, PAX5, and IRF4, may function as a stage-specific regulator during B cell development. Indeed, the genes commonly associated with these 4 TFs were highly enriched for B lymphocyte genes, such as *CD79A*, *CD79B*, and *PAX5* (Figure 2, D and E, and Supplemental Table 3). Consistently, transcript levels of those common targets were reduced upon depletion of each TF in MCL cells (Figure 2, F and G).

To gain further insight into how these TFs exert their function in MCL cells, we also performed a pathway analysis on genes occupied by either FOXO1 alone or FOXO1 together with EBF1, PAX5, or IRF4 (Supplemental Figure 3C). The analysis revealed that genes associated with the co-occupancies were enriched in pathways associated with B cell receptor (BCR) signaling, RNA metabolism, and apoptosis (Supplemental Figure 3D). To corroborate the findings from ChIP-Seq analysis, we next performed RNA-Seq analysis of CCMCL1 MCL cells at 96 hours after transduction of the sgRNA targeting each of the 4 TFs. The following pathway analysis identified hallmark gene sets, including interferon response and TNF/KRAS signaling, as the commonly affected pathways after TF depletion (Supplemental Figure 3E and Supplemental Table 4). Reactome gene set analysis further revealed that cell surface receptors such as BCR and immune signaling were other significantly affected pathways. Together, our findings indicate that the 4 TFs regulate an overlapping set of genes critical for MCL lineage viability.

### FOXO1 functions upstream of the MCL lineage regulatory TF hierarchy.

**Figure 3:**
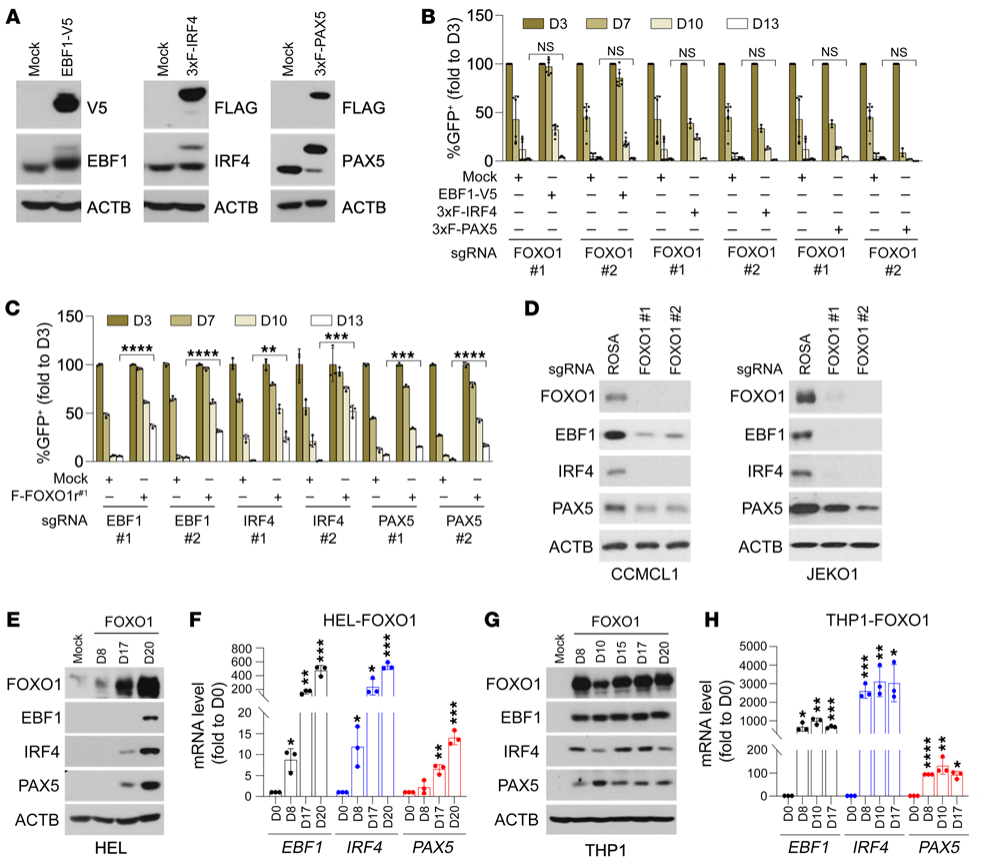
FOXO1 acts upstream of the MCL lineage regulatory TF hierarchy. (**A**) Immunoblot analysis of CCMCL1 cells transduced with EBF1-V5 (left), 3× FLAG–tagged IRF4 (middle), or 3× FLAG–tagged PAX5 (right). (**B**) Competition-based proliferation assays of sgRNAs against FOXO1 in Cas9-transduced CCMCL1 cells expressing mock control, EBF1-V5, or 3× FLAG–tagged IRF4 or PAX5. (**C**) Competition-based proliferation assays of sgRNAs against EBF1, IRF4, or PAX5 in control and FOXO1r^#1^-transduced (resistant for sgRNA#1 of FOXO1) CCMCL1 cells. In **B** and **C**, data represent mean ± SEM (*n* = 3). Results are representative of 3 independent experiments. Statistical analysis was performed using 2-tailed unpaired Student’s *t* test. The day 13 values of each cell line responding to the same sgRNA were respectively compared. **P < 0.001 ***P < 0.0005, ****P < 0.0001. (**D**) Immunoblot analysis of CCMCL1 (left) or JEKO1 (right) cells depleted of FOXO1. Lysates were prepared at day 3 after infection of indicated sgRNAs targeting FOXO1. (**E**–**H**) Immunoblot and RT-qPCR analysis of *EBF1*, *IRF4*, or *PAX5* induction in FOXO1-transduced HEL (**E** and **F**) or THP1 cells (**G** and **H**). Cell lysates and total RNA were prepared at indicated time points after infection of FOXO1-encoding lentivirus. In **F** and **H**, data represent mean ± SEM (*n* = 3). Results are representative of 3 independent experiments. Statistical analysis was performed using 1-way ANOVA with Tukey’s multiple-comparison test. *P < 0.05, **P < 0.005, ***P < 0.0005, ****P < 0.0001.

To explore the functional relationship among FOXO1, EBF1, IRF4, and PAX5, we next conducted competition-based proliferation assays to assess their mutual compensatory potential in MCL cell viability. Interestingly, while overexpression of EBF1, IRF4, or PAX5 in CCMCL1 cells did not affect sgFOXO1-induced growth arrest phenotype (Figure 3, A and B), enforced FOXO1 expression partially compensated for sgRNA-mediated EBF1, IRF4, or PAX5 depletion and enhanced cell survival in comparison with vector control–transduced CCMCL1 cells (Figure 3C). These findings raised the possibility that FOXO1 might function as an upstream regulator of the other 3 TFs. In agreement, immunoblot analysis of sgFOXO1-transduced CCMCL1 or JEKO1 cells revealed a markedly reduced expression of EBF1, IRF4, and PAX5 proteins in comparison with lentiviral sgROSA–infected control cells (Figure 3D).

FOXO1 is one of the pioneer factors that facilitate access to inactive chromatin by coregulator complexes (42, 43). To test whether enforced FOXO1 expression is sufficient to induce EBF1, IRF4, or PAX5 expression in non-B-lineage blood cancer cells, we next transduced lentivirus encoding FOXO1 into the AML cell lines HEL and THP1, which express negligible levels of EBF1, FOXO1, IRF4, and PAX5 (Supplemental Figure 4A). Strikingly, immunoblot and quantitative PCR with reverse transcription (RT-qPCR) analysis of FOXO1-transduced HEL or THP1 cells revealed a robust and time-dependent induction of EBF1, IRF4, and PAX5 expression, with eventual levels comparable to those observed in CCMCL1 cells (Figure 3, E–H). By contrast, ectopic expression of EBF1 (Supplemental Figure 4, B and C), IRF4 (Supplemental Figure 4, D and E), or PAX5 (Supplemental Figure 4, F and G) in HEL or THP1 cells could not reprogram the AML cells nor activate expression of the other MCL lineage-survival TFs. These findings together indicate that FOXO1 acts as an upstream TF to prime the expression of other MCL lineage-survival TFs. But despite its ability to reprogram AML cells, it is worthwhile to note that forced FOXO1 expression in HeLa or HEK295T cells of non-hematopoietic origin could not reprogram the cells nor promote other MCL lineage-survival TF expression (Supplemental Figure 4, H and I), implying that an appropriate cellular context is required for FOXO1 to carry out its lineage-priming function.

### A tiling scan reveals that the DNA binding and transactivation domains of FOXO1 are required for its MCL lineage-survival function.

**Figure 4:**
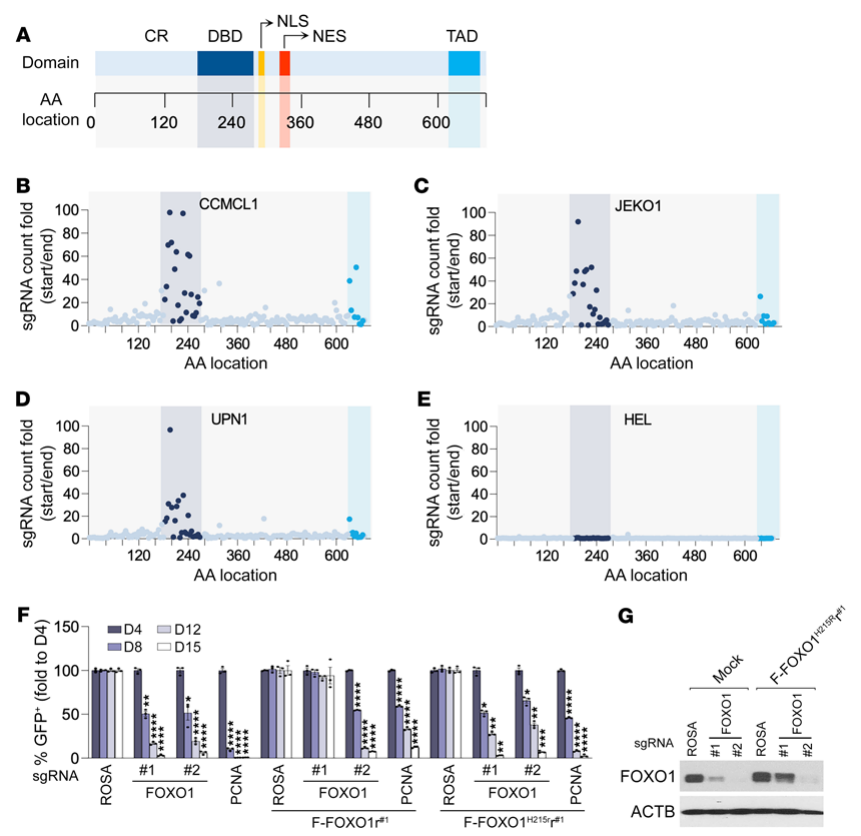
DBD and TAD of FOXO1 are required for its MCL lineage-survival function. (**A**) A schematic graph of structural domains of FOXO1 protein. CR, conserved region; DBD, DNA binding domain; TAD, transactivation domain; NLS, nuclear localization sequence; NES, nuclear export sequence. (**B**–**E**) Systematic evaluation of 167 FOXO1 sgRNAs in negative selection experiments. The location of each sgRNA relative to the FOXO1 protein is indicated along the *x* axis. The *y* axis is the fold change of the abundance of individual sgRNAs (ratio of start to end point) in Cas9-expressing CCMCL1 (**B**), JEKO1 (**C**), UPN1 (**D**), and HEL (**E**) cells after 14 population doublings. (**F**) Competition-based proliferation assays of sgRNAs against FOXO1 in Cas9-transduced CCMCL1 cells expressing wild-type FOXO1 (FOXO1r^#1^) or DNA binding–defective mutant (FOXO1^H215R^r^#1^). The FOXO1r^#1^ and FOXO1^H215R^r^#1^ mutants are CRISPR-resistant to the action of sgFOXO1#1 but sensitive to sgFOXO1#2. Data represent mean ± SEM (*n* = 3). Results are representative of 3 independent experiments. Statistical analysis was performed using 1-way ANOVA with Tukey’s multiple-comparison test. *P < 0.05, **P < 0.005, ***P < 0.0005, ****P < 0.0001. (**G**) Immunoblot analysis of control or FOXO1^H215R^r^#1^-expressing CCMCL1 cells at day 3 after transduction of indicated sgRNAs.

As a member of the forkhead family of TFs, FOXO1 consists of several structurally defined domains, including an N-terminal conserved region, a highly conserved forkhead DNA binding domain (DBD), a nuclear localization sequence, a nuclear export sequence, and a C-terminal transactivation domain (TAD) (Figure 4A) (44). To map the functional regions of FOXO1 that are critical for its MCL lineage-survival activity, we constructed a high-density CRISPR library of 167 sgRNAs that covered the entire open reading frame of human FOXO1 (Supplemental Table 5). A CRISPR-based tiling scan was carried out by introduction of the pooled FOXO1-targeting sgRNA library into the Cas9-expressing MCL (CCMCL1, JEKO1, and UPN1) or AML (HEL) cells. The relative impact of individual sgRNAs on cell growth over 14 population doublings was assessed via DNA sequencing–based quantification of sgRNA abundance. As predicted, the scan results confirmed that FOXO1 played an essential role in supporting MCL cell growth but was dispensable for HEL cells (Figure 4, B–E). But within the 167 sgRNAs designed to target different FOXO1 domains, we found a great variation in their ability to repress MCL cell propagation. All sgRNAs that induced more than 20-fold MCL depletion were clustered in the gene regions encoding the DBD and TAD motifs. By contrast, the sgRNAs targeting *FOXO1* regions encoding protein sequences outside of the DBD and TAD domains elicited minimal growth arrest phenotypes, supporting the notion that the severity of negative selection in CRISPR-mediated genome editing correlates with the functional importance of targeted motifs within a protein (45). These tiling scan results thus indicate that FOXO1’s transcriptional regulator activity is primarily responsible for its characteristic MCL-supporting function. Consistently, while complementation of a CRISPR-resistant wild-type FOXO1 cDNA readily rescued the sgFOXO1-induced dropout (Figure 1H and Figure 4F), the CCMCL1 cells transduced with a CRISPR-resistant DBD mutant (H215R) (46) failed to compensate for endogenous FOXO1 loss and remained highly sensitive to sgRNA-induced endogenous FOXO1 depletion (Figure 4, F and G), confirming that the transcriptional regulatory function of FOXO1 is necessary for its MCL lineage-survival function.

### The transactivation domain of FOXO1 specifies its lineage-survival TF activity in MCL cells.

**Figure 5:**
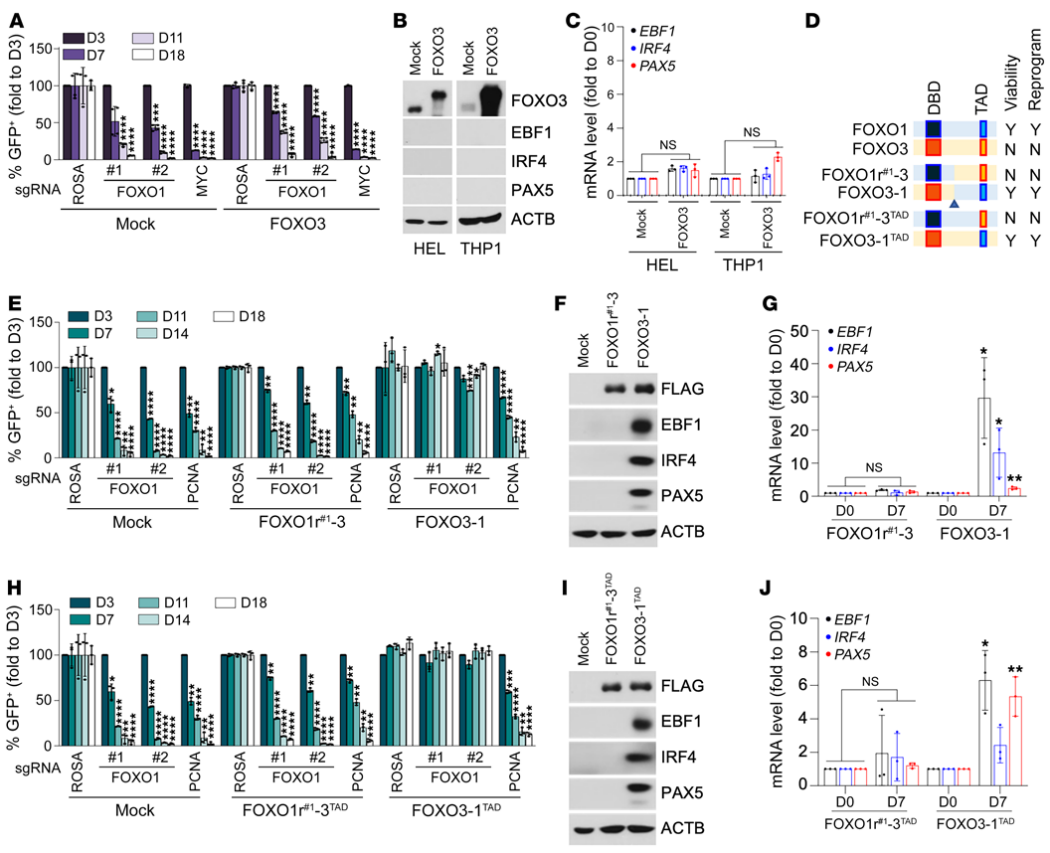
TAD of FOXO1 specifies its MCL lineage–supporting activity. (**A**) Competition-based proliferation assays for indicated FOXO1-targeted sgRNAs in mock control or FOXO3-transduced CCMCL1 cells. Data represent mean ± SEM (*n* = 4). (**B** and **C**) Immunoblot (**B**) and RT-qPCR analysis (**C**) of *EBF1*, *IRF4*, or *PAX5* expression in FOXO3-transduced HEL or THP1 cells. Cell lysates and total RNA were prepared at day 20 after infection of FOXO3-encoding lentivirus. Data represent mean ± SEM (*n* = 3). (**D**) A schematic of domain-swapped FOXO1 and FOXO3 mutants. N, no; Y, yes. (**E**) Competition-based proliferation assays for indicated FOXO1-targeted sgRNAs in FOXO1r-3–transduced (left) or FOXO3-1–transduced (right) CCMCL1 cells. Data represent mean ± SEM (*n* = 3). (**F** and **G**) Immunoblot (**F**) and RT-qPCR (**G**) analysis of *EBF1*, *IRF4*, and *PAX5* induction in FOXO1r^#1^-3–transduced (left) or FOXO3-1–transduced (right) THP1 cells. Cell lysates and total RNA were prepared at day 7 after infection of lentivirus encoding indicated variants. Data represent mean ± SEM (*n* = 3). (**H**) Competition-based proliferation assays in FOXO1r^#1^-3^TAD^–transduced (left) or FOXO3-1^TAD^–transduced (right) CCMCL1 cells. Data represent mean ± SEM (*n* = 3). (**I** and **J**) Immunoblot (**I**) and RT-qPCR (**J**) analysis of *EBF1*, *IRF4*, and *PAX5* induction in FOXO1r^#1^-3^TAD^–transduced (left) or FOXO3-1^TAD^–transduced (right) THP1 cells. The FOXO1r^#1^-3^TAD^ variant is CRISPR-resistant to the action of sgFOXO1#1 but remains sensitive to sgFOXO1#2. Data represent mean ± SEM (*n* = 3). (**A**, **C**, **E**, **G**, **H**, and **J**) Results are representative of 3 independent experiments. Statistical analysis was performed using 1-way ANOVA with Tukey’s multiple-comparison test in **A**, **E**, and **H** and using 2-tailed unpaired Student’s *t* test in **C**, **G**, and **J**. *P < 0.05, **P < 0.001, ***P < 0.0005, ****P < 0.0001.

FOXO1 belongs to the forkhead box O family of TFs, which are known for their highly homologous structures and functional redundancy (20). Although FOXO1 was identified as a crucial MCL lineage-survival TF, none of the other paralogs (i.e., FOXO3, FOXO4, and FOXO6) were scored in our CRISPR screen (Supplemental Figure 5A). Consistently, immunoblot analysis of a panel of MCL cell lines revealed that FOXO1, along with EBF1, IRF4, and PAX5, was highly expressed in MCL cells, in contrast to AML cells, which barely expressed any of the 4 MCL lineage-survival TFs (Supplemental Figure 4A and Supplemental Figure 5B). Moreover, among the FOXO paralogs, only FOXO1 was highly expressed in all MCL cell lines, while FOXO3 and FOXO4 expression was uneven across MCL cells and generally comparable to that in AML cells (Supplemental Figure 5, C–E and F). This raised the possibility that the selective requirement of FOXO1 as compared with its structurally and functionally homologous paralogs might be simply due to its predominant expression in MCL cells. To test this hypothesis, we next explored whether other FOXO paralogs could functionally substitute for FOXO1 in MCL cells. To our surprise, enforced expression of FOXO3, a highly homologous paralog of FOXO1, could not compensate for CRISPR-mediated FOXO1 depletion nor support the growth of CCMCL1 cells (Figure 5A and Supplemental Figure 6A). Consistently, overexpression of FOXO3, unlike enforced FOXO1 expression, did not elicit the B lineage–like reprogramming nor induce EBF1, PAX5, and IRF4 expression in the AML cell line HEL or THP1 (Figure 5, B and C). These findings indicate that FOXO1 is uniquely required for the growth of MCL-lineage cells.

FOXO1 and FOXO3 are highly structurally homologous proteins with a comparable length of approximately 660 amino acid residues. Sequence alignment indicated that the forkhead DBD and C-terminal TADs were among the regions showing the highest sequence conservation (Supplemental Figure 6B). Since ectopic FOXO3 expression could not compensate for the depletion of endogenous FOXO1 in supporting MCL cell growth, we next sought to determine the structural motif of FOXO1 that is responsible for this functional non-redundancy. We engineered 2 chimeric cDNA constructs by swapping the C-terminal sequences of FOXO1r^#1^ 1 (CRISPR-resistant synonymous mutant designed specifically for sgFOXO1r^#1^) and FOXO3 (Figure 5D). The sgRNA/cDNA rescue assays showed that ectopic expression of the FOXO3-1 variant containing the FLAG-tagged FOXO3 N-terminus (1–296 aa) and the FOXO1 C-terminus (299–655 aa) retained full functionality and could compensate for endogenous FOXO1 depletion in sgFOXO1-transduced CCMCL1 and UPN1 MCL cells (Figure 5E and Supplemental Figure 6, C–E). By contrast, transduction of the FOXO1r^#1^-3 variant harboring N-terminal FOXO1r^#1^ (1–298 aa) and C-terminal FOXO3 (297–673 aa) could not rescue the growth arrest phenotype of CCMCL1 and UPN1 cells upon sgRNA-mediated endogenous FOXO1 depletion. Consistently, immunoblot and RT-qPCR analyses of the AML cell line THP1 revealed that enforced expression of FOXO3-1, but not the FOXO1r^#1^-3 variant, was able to robustly induce MCL lineage-survival TF expression in AML-line THP1 cells (Figure 5, F and G, and Supplemental Figure 6, F and G). These findings indicate that the functional determinant of the FOXO1 specificity resides within the C-terminal regions.

Since our tiling scan identified that the TAD motif within the FOXO1 C-terminal regions is essential for MCL growth, we next performed a domain-swap assay by constructing a FOXO3 TAD–harboring FOXO1 variant resistant to sgFOXO1^#1^ (hereafter referred to as FOXO1r^#1^-3^TAD^) and a FOXO3 variant swapped for FOXO1 TAD (referred to as FOXO3-1^TAD^). Interestingly, although 4XIRE-luciferase reporter assays showed that these constructs had comparable transcriptional transactivation activity on FOXO-driven reporter activity (Supplemental Figure 6H), ectopic expression of FOXO1r^#1^-3^TAD^ could not rescue sgFOXO1-transduced CCMCL1 cells. By contrast, FOXO3-1^TAD^ expression fully compensated for the endogenous FOXO1 depletion and rendered the resistance to FOXO1-targeted sgRNAs (Figure 5H and Supplemental Figure 6, I–K). Consistently, immunoblot and RT-qPCR analyses showed that ectopic expression of FOXO3-1^TAD^, but not the FOXO1r^#1^-3^TAD^ variant, induced a robust expression of EBF1 and PAX5 in THP1 cells (Figure 5, I and J, and Supplemental Figure 6, L and M). These findings together establish the TAD motif as the determinant of FOXO1’s specific lineage-supporting function.

### Cpd10 is a FOXO1-specific inhibitor that suppresses MCL growth in vitro.

**Figure 6:**
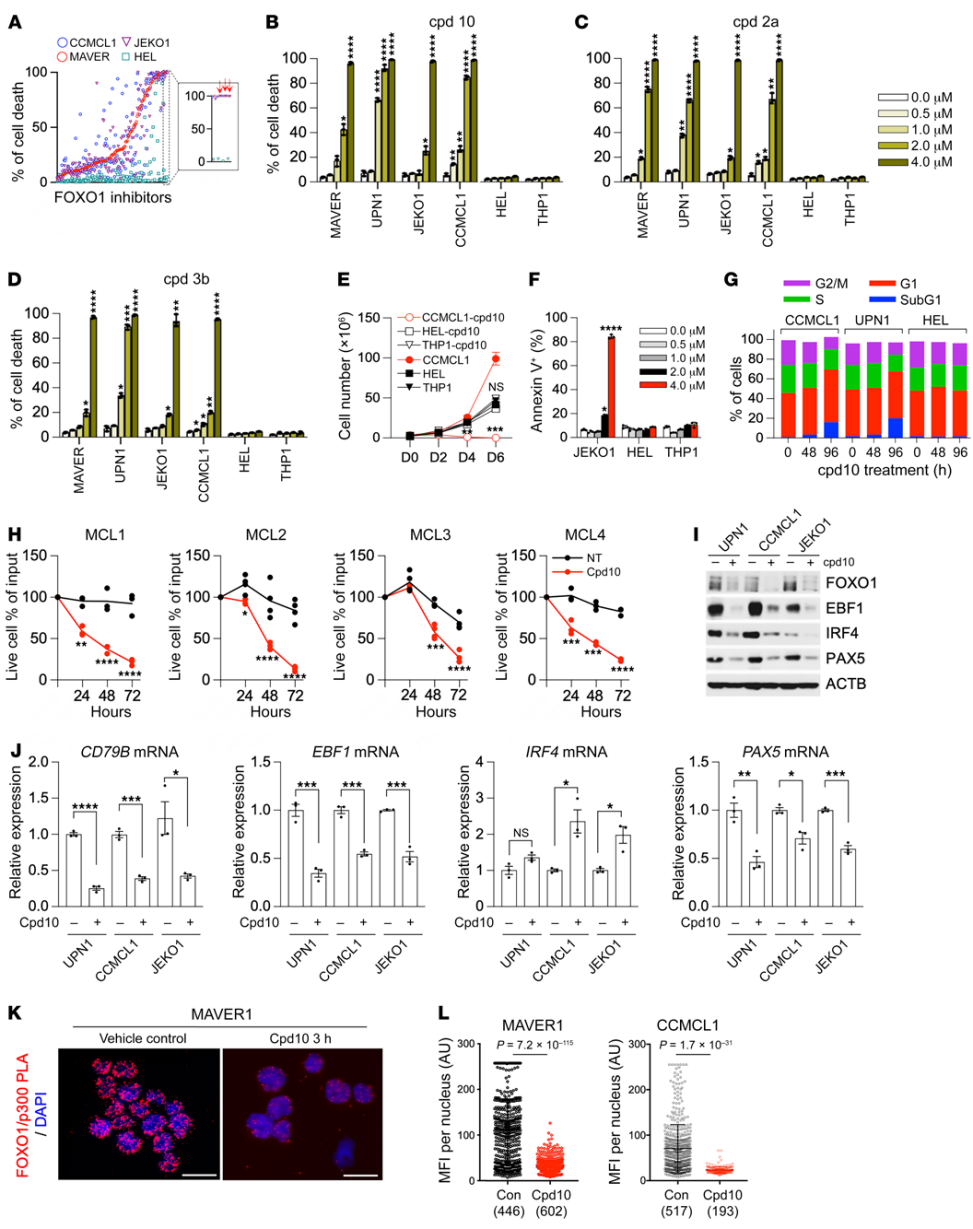
Cpd10 is a FOXO1-specific inhibitor that suppresses MCL growth in vitro. (**A**) Ranking of cell death induction activity of 144 FOXO1 small-molecule inhibitors. Cell death was measured by TO-PRO-3 cell death assay (Thermo Fisher Scientific) after 6-day treatment of compounds (4 μM) in 4 indicated cell lines. Red arrows point to the selected inhibitors for follow-up studies. (**B**–**D**) Dose-dependent effect of FOXO1 inhibitors on MCL and AML cell lines. Relative cell death (percent) was determined by TO-PRO-3 staining at day 6 under treatment of the indicated compounds. (**E**) Growth curve of CCMCL1, HEL, and THP1 cells under cpd10 (2 μM). (**F**) Percentage of annexin V–positive JEKO1, HEL, and THP1 cells at day 6 following treatment with cpd10 (2 μM). (**G**) Cell cycle distribution of cells treated with cpd10 (2 μM). (**H**) Primary MCL cells were cultured with cpd10 (2 μM) or vehicle. Data represent mean ± SEM (*n* = 4, MCL1, MCL4; *n* = 3, MCL2, MCL3). Results are representative of 2 independent experiments. (**I** and **J**) Immunoblot (**I**) and RT-qPCR assay (**J**) of *CD79B*, *EBF1*, *IRF4*, or *PAX5* mRNA expression in 3 indicated control and cpd10-treated cell lines. Total RNAs were prepared after 48 hours of treatment. (**K**) Representative proximity ligation assay (PLA) image of MAVER1 cells demonstrating the inhibition of interaction between FOXO1 and p300 in response to 2 μM cpd10. Scale bars: 20 μm. (**L**) Quantitation of mean fluorescence intensity (MFI) of PLA signal per nuclei from MAVER1 or CCMCL1 cells. The number of nuclei scored is indicated. (**B**–**F**, **J**, and **L**) Data represent mean ± SEM (*n* = 3). Results are representative of 3 independent experiments. Statistical analysis was performed using 1-way ANOVA with Tukey’s multiple-comparison test in **B**–**D** and using 2-tailed unpaired Student’s *t* test in **E**, **F**, **H**, **J**, and **L**. *P < 0.05, **P < 0.001, ***P < 0.0005, ****P < 0.0001.

FOXO1 inhibitors, particularly AS1842856, have been previously used in studies of B cell precursor acute lymphoblastic leukemia (BCP-ALL) (47) and BL (48, 49). But despite its visible efficacy, its selectivity profile and off-target effects have been noted (50). To identify FOXO1-specific inhibitors with potent cytotoxic effects in MCL, we leveraged a FOXO1-focused investigational small-molecule compound library (51). These 144 compounds were all found to have at least 10-fold selectivity for FOXO1 over FOXA2 based on reporter gene assays and were curated against general cytotoxicity up to 50 μM concentration. Using a single-dose (4 μM) triage strategy, we pared down to the top 20 compounds by testing their potencies in 3 MCL cell lines (MAVER1, JEKO1, and CCMCL1) and counterscreening in the AML cell line HEL (Figure 6A). Compounds that consistently induced a cytotoxic response across all MCL cells but elicited only baseline cytotoxicity in the control AML cells were prioritized for further analyses. Based on their single-dose activities, 3 top hits — cpd10, cpd2a, and cpd3b — were selected for dose-response studies (Figure 6, B–D). Among them, cpd10 (*N*-[3-(1*H*-1,3-benzodiazol-2-yl)-1*H*-pyrazol-5-yl]-4-(4-methylpiperazin-1-yl)benzamide) is a selective FOXO1 inhibitor whose in vivo specificity and activity we previously demonstrated (Supplemental Figure 7A) (50). Importantly, cpd10 treatment (2 μM) significantly suppressed the growth of MCL cell lines JEKO1 and CCMCL1 but showed little effect on the proliferation of AML cell lines HEL and THP1 (Figure 6E). Cell cycle and cell death analysis revealed a notable increase of sub-G_1_ fraction and annexin V–positive apoptotic cell population in cpd10-treated MCL but not AML cells, as compared with their respective mock-treated control cells (Figure 6, F and G). We further tested cpd10 sensitivity in 4 primary MCL cells. Consistent with the results from MCL cell lines, cpd10 treatment (2 μM) clearly inhibited the survival of primary MCL cells (Figure 6H).

In agreement with our screen results, 4XIRE reporter assays of cpd10 confirmed its specific FOXO1 inhibition (IC_50_ < 0.1 μM). Treatment of cells expressing wild-type FOXO1 with cpd10 led to a dose-dependent reduction of FOXO reporter activity but had no effect on FOXA2-driven reporter (Supplemental Figure 7B). Consistently, immunoblot and RT-qPCR analysis of cpd10-treated MCL cells revealed a significant reduction of expression of the FOXO1 target genes *EBF1*, *PAX5*, and *CD79B* in comparison with mock-treated control cells (Figure 6, I and J). Interestingly, IRF4 was downregulated only at the protein level, suggesting it might not be a direct transcriptional target of FOXO1. Although prolonged cpd10 treatment led to FOXO1 degradation, acute cpd10 treatment did not interfere with FOXO1 binding to its target regulatory loci, *TXNIP* or *CD79A*, as determined by ChIP–RT-qPCR (Supplemental Figure 7C). Instead, we found that cpd10 treatment disrupted the interaction of FOXO1 with its coactivator p300, as evidenced by the diminished coimmunoprecipitation (Supplemental Figure 7D) and loss of nuclear FOXO1/p300 interaction by proximity ligation assay in comparison with the mock-treated controls (Figure 6, K and L). Consistently, H3K27ac ChIP-Seq analysis revealed that genetic FOXO1 knockdown and cpd10 treatment in CCMCL1 cells both diminished H3K27ac peak intensity on FOXO1 peak-centered loci in comparison with the mock-treated control cells (Supplemental Figure 7, E and F). RNA-Seq analysis confirmed the coclustering of cpd10-treated and genetically FOXO1-inhibited JEKO1 and CCMCL1 as opposed to their mock-treated control cells (Supplemental Figure 7G). Further pathway enrichment analysis of differentially expressed genes revealed common gene sets (i.e., KRAS, TNF, or interferon signaling) in both JEKO1 and CCMCL1 cells (Supplemental Figure 7H). These results together support that cpd10 is a FOXO1 inhibitor that works by repressing coactivator recruitment.

### Pharmacological targeting of FOXO1 suppresses MCL progression in vivo.

We next investigated the relevance of FOXO1 inhibition to MCL progression in vivo. CCMCL1 is an MCL line derived from engrafted patient-derived tumor cells that were passaged in NSG (NOD.Cg-PrkdcscidIl2rgtm1Wjl/SzJ) mice (52). As a well-characterized and reliable model, CCMCL1 cells have been routinely used for in vivo MCL preclinical studies. To assess the impact of FOXO1 inhibition on MCL growth in vivo, control and FOXO1-targeted sgRNA-transduced CCMCL1 cells were transplanted via tail vein injection into secondary recipient NSG mice. As determined by bioluminescence imaging (BLI), the control mice with 2 × 10^6^ transplanted sgROSA-CCMCL1 cells generally developed tumors within a week after inoculation. By day 21, all control mice succumbed to the outgrowth of tumor cells (Supplemental Figure 7, I–K). By comparison, depletion of FOXO1 by sgFOXO1 resulted in a marked delay in MCL progression and a survival benefit as evidenced by the diminished tumor burden and spleen enlargement (Supplemental Figure 7L), confirming that FOXO1 inhibition could suppress MCL progression in vivo.

**Figure 7:**
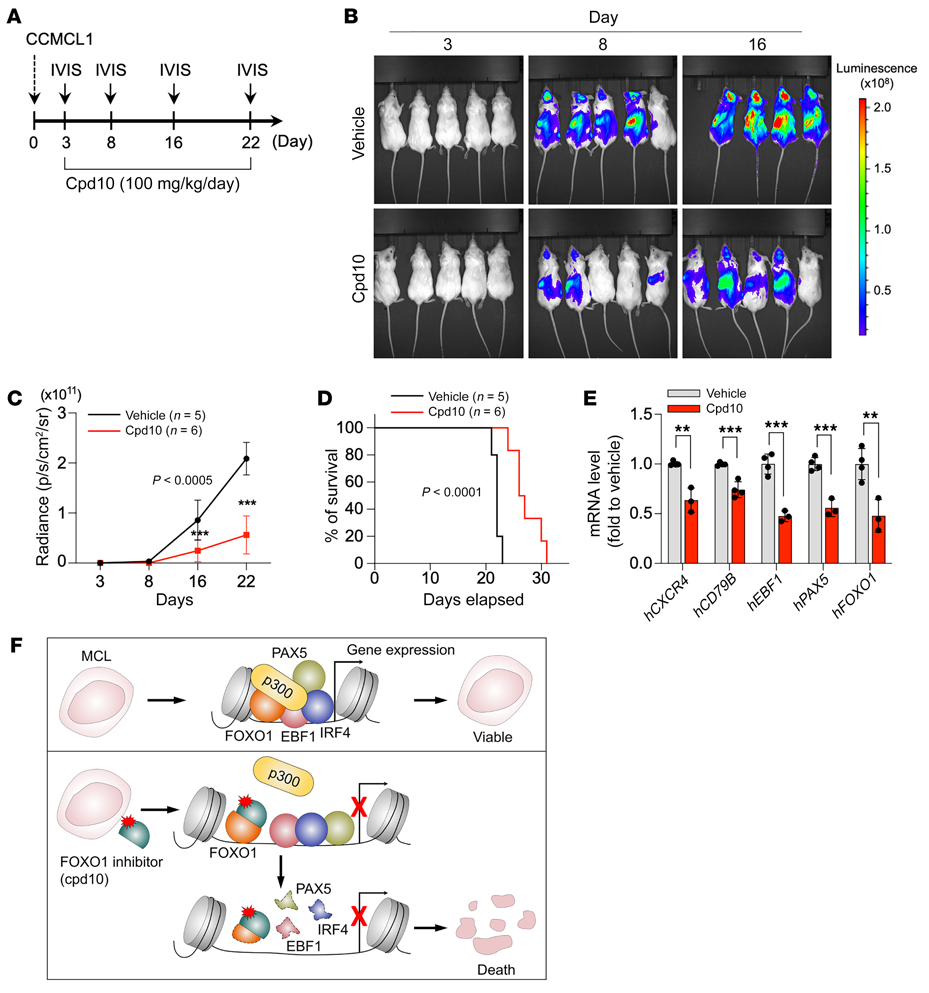
Pharmacological inhibition of FOXO1 suppresses MCL progression in vivo. (**A**) Experimental design of the in vivo treatment. (**B**) Bioluminescent imaging of CCMCL1 MCL recipient mice at the indicated day after initiation of treatment with cpd10 (100 mg/kg/d) or vehicle control. (**C**) Quantification of bioluminescent imaging responses to cpd10 treatment. Mean values of vehicle-treated (*n* = 5) and cpd10-treated (*n* = 6) mice are shown. Data represent mean ± SEM (*n* = 5 or 6). Results are representative of 2 independent experiments. Statistical analysis was performed using 2-tailed unpaired Student’s *t* test. ***P < 0.0005. (**D**) Kaplan-Meier survival curves of control and cpd10-treated mice. Statistical significance was determined using a log-rank test. (**E**) RT-qPCR analysis of indicated human genes in vehicle- and cpd10-treated spleens of CCMCL1 MCL recipient mice. Data represent mean ± SEM (*n* = 3 or 4). Results are representative of 2 independent experiments. Statistical analysis was performed using 2-tailed unpaired Student’s *t* test. **P < 0.005, ***P < 0.0005. (**F**) Predicted model of MCL lineage-survival transcriptional program and its dissolution following targeted inhibition of FOXO1.

We then proceeded to examine the pharmacological antitumor activity of the FOXO1 inhibitor cpd10. A pharmacokinetic assessment indicated that cpd10 is a well-tolerated compound with significant plasma exposure by multiple routes of administration and suitable for in vivo work (Supplemental Table 6). To test whether cpd10 has single-agent activity in established MCL, NSG mice underwent transplantation of CCMCL1 cells by tail vein injection and were monitored by BLI. Upon detection of the disease on day 3 following inoculation, the MCL-bearing mice were randomly grouped and dosed once daily with cpd10 (100 mg/kg per dose, i.p.) or vehicle. Remarkably, cpd10 administration led to a substantial delay in disease progression and significantly extended survival (Figure 7, A–D). The cpd10 treatment was well tolerated in the experimental group of C57BL/6J mice, with no visible body weight loss and only marginal impact on normal B cell content (Supplemental Figure 7, M and N). Moreover, analysis of spleen tissues from cpd10-treated animal groups revealed a reduced expression of the FOXO1 transcriptional targets *CXCR4*, *CD79B*, *PAX5*, and *EBF1* (Figure 7E). Together, these findings indicate that cpd10 has efficacious anti-MCL activity as a single agent in vivo.

## Discussion

It has been increasingly appreciated that oncogenic events occur in the context of cellular identity. As a result, genetic or pharmacological inhibition of the core lineage-specific TFs that implement cancer cell identities may impair tumor viability by disrupting their gene expression networks (13, 14). In this study, we have identified FOXO1, EBF1, PAX5, and IRF4 as 4 critical lineage-survival TFs in MCL. Among these, FOXO1 is sufficient to drive the MCL lineage transcription program by supporting the expression of other lineage-survival TFs. We further show that FOXO1, but not its closely related paralogs, is selectively required for MCL lineage maintenance. The domain-swap assay pinpoints the transactivation domain (TAD) of FOXO1 as the determinant of the dedicated function. Finally, we have identified cpd10 as a FOXO1-targeted lead compound and verified that cpd10 treatment of MCL cells elicits a robust cytotoxic response in vitro and suppresses tumor progression in vivo. These findings identify FOXO1 as a master regulator for MCL lineage survival and highlight pharmacological FOXO1 inhibition as a therapeutic strategy targeting lineage-driven transcriptional addiction in MCL (Figure 7F).

Many tumors rely crucially on the proliferation and survival programs that are embedded within normal lineage precursor cells through development. Thus, tumor lineage-survival TFs that support the tumor transcriptional networks represent a large class of non-oncogene dependencies and potential therapeutic targets (14). Indeed, the 4 MCL lineage-survival TFs identified in our CRISPR screen had been previously implicated in early B cell development (40), likely reflective of the origin of MCL as a mature B cell malignancy from pre-germinal-center-stage B cells. During early B cell development, FOXO1 expression is induced in common lymphoid progenitors by the action of E2A and HEB proteins to specify B cell fate. The induced FOXO1 then acts in concert with E2A to activate the expression of EBF1, PAX5, and IRF4 to fully establish the B-lineage gene expression program (53, 54). This is consistent with our findings that FOXO1 acts in the upstream regulatory hierarchy of MCL lineage commitment and expression. Moreover, our ChIP-Seq analysis reveals that the annotated gene regulatory regions of the other 3 TFs were highly overlapped with those of FOXO1, suggesting that their coordinated activity is necessary for establishing MCL transcriptional identity and supporting tumor cell survival. Notably, despite their upstream regulatory role in B-lineage commitment, our CRISPR screen reveals that E2A and HEB are dispensable for MCL propagation (Figure 1C), suggesting that MCL lineage-survival TF expression no longer depends on the early B lineage–specifying TFs.

Our findings, together with earlier studies, suggest that FOXO1 is required for B cell–originating tumor survival. In addition to MCL cells, FOXO1 dependency has been found in BCP-ALL (47) and BL (48, 49). By comparison, we found that germinal center–derived DLBCL and BL cells exhibited remarkably less dependency on FOXO1 despite their mature B cell origin (Supplemental Figure 2, E–G), suggesting that extragenetic or epigenetic alterations may substitute for the FOXO1 lineage-survival program in different cellular contexts. In line with this idea, ectopic expression of the FOXO1 downstream effector CCND3 largely rescued BCP-ALL cells from FOXO1 inactivation–induced growth arrest and apoptosis (47). However, overexpression of CCND1, a predominant D-type cyclin of MCL, could not protect MCL cells from FOXO1 depletion–induced cell death, indicating the presence of distinct FOXO1 lineage-survival programs. Future investigation is needed to determine the detailed molecular events that follow FOXO1 action in different subsets of B cell–originated malignancies.

As one of the effector arms of PI3K/AKT signaling, FOXO TFs have been implicated in a wide variety of biological processes, including tumor suppression. We and others previously showed that mice somatically depleted of FOXO1 along with its 2 close paralogs, FOXO3 and FOXO4, are predisposed to the development of thymic lymphomas and hemangiomas (20). In contrast to those well-described tumor-suppressive activities, the current study reveals that FOXO1 may also act as a pro-oncogenic lineage-survival TF in MCL. Genetic or pharmacological inhibition of FOXO1 collapses the MCL transcriptional network and induces tumor cell death in vitro and in vivo. These observations underscore the highly context-dependent nature of FOXO1 functions in tumorigenesis. Consistent with our findings, somatic missense FOXO1 mutations that target its AKT recognition motif have been recurrently identified in a significant fraction of germinal center origin of B cell lymphomas, including DLBCL, follicular lymphomas, and BL (29, 30, 55–58). Moreover, studies in BL and B cell non-Hodgkin’s lymphomas have demonstrated FOXO1 mutation as a pro-oncogenic event in germinal center B cell–derived lymphomas owing to its pro-proliferative and antiapoptotic activity (59, 60). Interestingly, although somatic gain-of-function hotspot FOXO1 mutations have not been found in MCL, our examination of human MCL cell lines found a frequent coexistence of high levels of FOXO1 protein expression with hyperactivated PI3K/AKT signaling (Supplemental Figure 5B), suggesting that FOXO1 protein stability and PI3K/AKT activity are likely uncoupled in MCL.

FOXO TFs are an evolutionarily conserved family of transcriptional regulators that includes 3 highly related paralogs (FOXO1, FOXO3, and FOXO4) with overlapping patterns of expression and transcriptional activities and a fourth more distantly related member (FOXO6) regulated by distinct mechanisms (61). FOXO1, FOXO3, and FOXO4 behave in a highly similar manner in biochemical studies and regulate a common set of target genes through a shared core recognition motif, [A/G]TAAA[T/C]A (62). Given their extensive functional redundancies, it is unanticipated that only FOXO1, among the 4 family members, is specifically required for MCL survival. This dedicated function of FOXO1 is clearly not due to its distinct tissue- or lineage-specific expression patterns or regulatory mechanisms, as ectopic expression of its close relative FOXO3 fails to rescue the FOXO1 depletion–induced growth arrest phenotype in MCL cells. Our domain-swap analysis further successfully narrowed down the underlying structural determinant to the TAD of FOXO1. But despite these findings, we still have not been able to pinpoint the underlying molecular mechanism. Hence, an important area of future study will be to determine the other TF(s) or cofactor(s) that cooperate with FOXO1 to carry out its dedicated MCL lineage-survival TF function.

Precision oncology efforts have led to the development of new therapies for the treatment of refractory and resistant MCL, such as CD19 CAR T cells and non-covalent inhibitors of BTK (6–11). However, despite initial success, new therapeutic targets and strategies are needed to circumvent the resistance. Thus, the development of therapeutic agents that target the MCL lineage dependency through FOXO1 inhibition may meet the task by not only expanding the targeted approaches but also providing complementary agents for future combination regimens. Targeting lineage-survival TFs raises the possibility of on-target toxicity. For example, pharmacological inhibition of FOXO1 may theoretically also affect normal B cell development based on its mechanism of action. Despite those potential concerns, we expect that the side effect will be manageable with carefully scheduled dosing and the effect will likely be reversible upon cessation of the treatment, as with the CD19 CAR T cell therapeutics (63). By executing a small-molecule screen of a selective compound library, we have identified cpd10 as a FOXO1-targeted lead compound. We further validated that cpd10 treatment inhibits MCL cell propagation in vitro and tumor growth in vivo. Importantly, the treatment was well tolerated in our study. We found no overt toxicity in the experimental animals that had been given cpd10 (100 mg/kg per dose per day, i.p.) for over a month. The findings support that pharmacological inhibition of lineage-survival TFs may be used as a therapeutic strategy to target lineage-driven transcriptional addiction in MCL.

## Methods

Information regarding the usage of antibodies, bacterial strains, chemical and biological agents, cell lines, primers, recombinant DNA, software, algorithms, and instruments is included in Supplemental Table 7.

### Cell lines and culture details.

MCL cell lines CCMCL1, JEKO1, UPN1, MAVER, MINO, Z138, SP53, and REC1, leukemia cell lines HEL, THP1, and K562, and BL lines BJAB and DG75 were cultured in RPMI 1640 medium supplemented with 10% heat-inactivated FBS, 2 M l-glutamine, and 50 U/mL penicillin-streptomycin. Human cell lines H1299, U2OS, HeLa, and HEK293T were maintained in DMEM supplemented with 10% FBS and 50 U/mL penicillin-streptomycin at 37°C in a humidified 5% CO_2_ incubator. All cell lines were lentivirally transduced to express human codon-optimized SpCas9 nuclease for establishment of somatic-deletion cells by CRISPR/Cas9 genome editing. Infected cells were selected with blasticidin or puromycin for 7–14 days. All cell lines were repeatedly tested for mycoplasma contamination by PCR.

### Primary MCL cell culture.

Peripheral blood from MCL patients was obtained following written informed consent under a protocol approved by the Institutional Review Board of The Ohio State University in accordance with the Declaration of Helsinki. Non-nodal leukemic MCL cases were excluded. After thawing of cryopreserved peripheral blood mononuclear cells according to standard protocols, MCL cells were isolated using CD19 magnetic beads and cultured according to standard methods. Purity of the isolated MCL cells was determined by flow cytometry analysis using CD45, CD5, and CD20 staining. Patient characteristics are included in Supplemental Table 2. Primary MCL cells were cultured as previously described with modifications (64). Primary MCL cells were cultured in RPMI 1640 medium supplemented with 20% FBS, 50 U/mL penicillin-streptomycin, 2 mM l-glutamine, IL-6 (40 U/mL), soluble IL-6R (40 U/mL), IGF-1 (30 ng/mL), sCD40L (0.5 μg/mL), and BAFF (50 ng/mL) and cocultured with an HS-5 (GFP-positive) stromal cell layer.

### Treatment of MCL xenografts.

Mice were maintained on a 12-hour light/12-hour dark cycle, and food and water were provided ad libitum. Mice of either sex were used. For in vivo transplantation of MCL cells, CCMCL1 cells expressing Cas9 and luciferase were infected with sgFOXO1 or sgROSA. On day 3 after infection with the sgRNA, the infection rate was checked by the percentage of GFP-positive cells, and all samples had a more than 90% infection rate. One million cells were injected i.v. into 8- to 12-week-old NSG mice (The Jackson Laboratory, 5557). To detect the disease progression, mice were imaged with an IVIS Spectrum system (Caliper Life Sciences).

For cpd10 treatment, NSG mice were injected i.v. with 2 × 10^6^ CCMCL1 cells. Mice were randomized based on in vivo imaging system (IVIS) signal, and treatment started on day 4. Mice were treated i.p. with vehicle or cpd10 (100 mg/kg daily). Cpd10 was formulated in Solutol HS-15/saline (5:95 vol/vol). Disease progression was monitored by IVIS scanning every 5 days.

### Plasmid construction and sgRNA cloning.

All the sgRNAs targeting human genes were cloned into either LRG2.1 or lenticrisprV2 following the protocol of Feng Zhang and colleagues (65). In brief, sgRNAs were cloned by annealing of 2 DNA oligonucleotides and T4 DNA ligation into a BsmB1-digested LRG2.1 or lenticrisprV2 vector.

For the cDNA overexpression experiments, a full-length *FOXO1*, *FOXO3*, *PAX5*, *EBF1*, and *IRF4* cDNA and each 3× N-terminal FLAG were amplified using CloneAmp HiFi PCR Premix (Takara Bio). FLAG and cDNA templates were cloned into pLu, pLx304, or pBabe vector using Gibson Assembly Master Mix (New England Biolabs). After ligation, plasmid DNA was transformed into Stbl3. Further information about the reagent and the primers for cloning is listed in Supplemental Table 7.

### Domain-focused sgRNA pooled library.

sgRNA pooled library TFs were provided by Christopher Vakoc, and this experiment followed the method of his group’s previous study (34). sgRNA oligonucleotides for *FOXO1* were selected based on domain information, synthesized in a pooled format (Integrated DNA Technologies), and then amplified by PCR using CloneAmp HiFi PCR Premix. PCR-amplified products were cloned into BsmB1-digested LRG2.1 vector using Gibson Assembly Master Mix. After ligation, plasmid DNA was transformed into Stbl3 and cultured overnight at 37°C. The plasmid DNA was extracted with a NucleoBond Xtra Maxi kit (Macherey-Nagel).

### Competition-based proliferation assays (GFP dropout assay).

Cas9-expressing cell lines were infected with sgRNAs linked with GFP using the U6-sgRNA-GFP plasmid. For dropout assay, cells were seeded at a density of 0.25 × 10^6^ in a 6-well culture plate and infected with the indicated sgRNAs. After 3 or 4 days of transduction, 75% of total cells were harvested, and then flow cytometry analysis was performed. The percentage of GFP-positive cell population was analyzed time-dependently with an LSR II flow cytometer (BD Biosciences). To assess the impact of individual sgRNAs on cellular proliferation, percentage GFP-positive at the final time point was compared with that at day 3 or 4 after transduction.

### Generation of viral particles.

Viruses were produced by cotransfection of indicated plasmids and packaging vectors into HEK293T packaging cells as previously described (66). In brief, to generate lentivirus, 8 × 10^6^ 293T cells in 100 mm tissue culture dishes were transfected with 8.5 μg of each plasmid DNA along with 4 μg of pMD2.G and 6 μg of psPAX2 packaging vectors using polyethylenimine. To produce retrovirus, 10 μg of each plasmid DNA and 10 μg of amphotropic vector were transfected into HEK293T cells.

### sgRNA pooled library negative selection screening.

Each MCL culture that expressed Cas9 stably was infected at 40% to avoid multiple sgRNA infection. A minimum of 10^8^ cells were kept to maintain the representation of all sgRNAs in the library. At day 3 after infection, 10^8^ cells were collected and used as a reference representation of the library. Cells were further cultured for 20 population doublings and collected. The collected cells were lysed with DNA extraction buffer (10 mM Tris-HCl, 150 mM NaCl, 10 mM EDTA, and 0.2% SDS) including proteinase K (100 mg/mL) overnight at 56°C. Genomic DNA was extracted by 3 rounds of phenol and precipitated in 75% ethanol overnight at –20°C. The precipitated pellet was washed with cold 75% EtOH and resolved with water for 1 hour at 56°C. To amplify the sgRNA library, a PCR reaction was performed for each genomic DNA using Takara Taq enzyme. The PCR product was collected, and about 250 bp of DNA was extracted using gel electroporation. Twenty nanograms of first PCR product was run to ligate the barcode for sequencing using Q5 High-Fidelity 2× Master Mix (New England Biolabs).

### RNA extraction and RT-qPCR.

Total RNAs were extracted from cells using a NucleoSpin RNA kit (Macherey-Nagel). Reverse transcription was carried out on 1.0 μg of total RNA using a RevertAid RT kit (Thermo Fisher Scientific). RT-qPCR was performed on cDNA samples using the PowerUp SYBR Green Master Mix (Thermo Fisher Scientific) on the 7500 Fast Real-time PCR system (Thermo Fisher Scientific). All samples were run in triplicate, and the mRNA level of each sample was normalized to that of *ACTB* mRNA. The relative mRNA level was presented as unit values of 2^ΔΔCt^.

### Immunoblot analysis.

Cells were lysed by 1× Laemmli buffer followed by sonication (30 watts/5 seconds/10 cycles). Protein concentration was determined using Pierce BCA protein assay kit. One to thirty micrograms of proteins were separated by SDS-PAGE electrophoresis and transferred to PVDF membrane, which was incubated with primary antibodies against ACTB (1:10,000), V5 (1:3,000), FLAG (1:1,000), EBF1 (1:1,000), FOXO1 (1:1,000), FOXO3 (1:1,000), IRF4 (1:1,000), PAX5 (1:1,000), AKT (1:1,000), p-AKT (S473) (1:1,000), or TUBA (1:10,000) overnight at 4°C and then with HRP-conjugated anti-mouse or anti-rabbit IgG. Blots were developed with the SuperSignal West Pico Chemiluminescent substrate (Thermo Fisher Scientific). Further information about the usage of antibodies is included in Supplemental Table 7.

### Coimmunoprecipitation assay.

Coimmunoprecipitation assay was performed as previously described (67). In brief, cells were lysed following the protocol of Rubio et al. Protein extracts (0.8 mg) were incubated with anti-FLAG magnetic beads with normal IgG as a control for 12 hours at 4°C.

### Proximity ligation assay.

Cells were attached to a glass slide using a cytospin centrifuge, fixed with 4% paraformaldehyde, and permeabilized in 0.2% Triton X-100. Primary antibodies (FOXO1, 1:500, Abcam, catalog 39670; and p300, 1:100, Sigma-Aldrich, catalog SAB1400094 clone 1B1) were incubated overnight at 4°C. Detection was performed using the Duolink In Situ Red kit (Sigma-Aldrich, catalog DUO92101) according to the manufacturer’s instruction. Images were acquired with an EVOS Auto FL microscope system using a ×40 objective. Mean fluorescence intensity per nucleus was determined using ImageJ 1.52k (NIH) software.

### RNA sequencing and data analysis.

Total RNAs were isolated from cells and subjected to RNA sequencing at the Genomics Resources Core facility of Weill Cornell Medicine. RNA-Seq libraries were prepared using the Illumina TruSeq stranded mRNA library preparation kit and sequenced on a HiSeq4000 sequencer (Illumina). Before analysis, library quality was assessed using FastQC (https://www.bioinformatics.babraham.ac.uk/projects/fastqc/) and adapters/sequencing artifacts were removed using Cutadapt (v1.8.2) (68) for all data. Next, gene expression quantification was accessed with selective alignment and fragment/GC content bias correction against the GENCODE 41 human transcript annotations, using salmon (v1.6.0) (69). The expression counts were normalized by the trimmed mean of M values method (70) and transformed to counts per million. The genes were ranked by the ratio between the knockouts (average of each target gene) and their respective controls and used to perform functional pathway enrichment via gene set enrichment analysis (GSEA) using a Monte Carlo adaptive multilevel splitting approach, implemented in the fgsea package (71). The hallmark collection obtained from the Broad Institute MSigDB database (72) was used for the analysis.

### ChIP and ChIP-qPCR.

ChIP analysis was performed as described previously (73). In brief, 3 × 10^7^ cells were sequentially cross-linked with 5 mM ethylene glycol bis(succinimidyl succinate) (EGS[Thermo Fisher Scientific]) for 45 minutes and with 1% paraformaldehyde for 10 minutes and quenched with 120 mM glycine for 5 minutes at room temperature. After nucleus isolation, the chromatin was digested with 6,000 gel units of micrococcal nuclease for 10 minutes at 37°C, followed by sonication in shearing buffer (50 mM Tris-HCl, 10 mM EDTA, and 0.1% SDS) using the Covaris M220 Focused ultrasonicator. Immunoprecipitation was performed with 10 μg of anti-FOXO1 overnight at 4°C. Twenty microliters of precleared Dynabeads Protein G (Thermo Fisher Scientific) was added and incubated for 3 hours at 4°C. Beads were washed by high-salt buffer (50 mM HEPES, 500 mM NaCl, 1 mM EDTA, 0.1% SDS, 1% Triton X-100, and 0.1% sodium deoxycholate) and RIPA buffer (including LiCl). The chromatin was eluted in elution buffer (50 mM Tris-HCl, 10 mM EDTA, and 1% SDS) and reverse cross-linked at 65°C overnight. DNA was extracted using NucleoSpin Gel and PCR clean-up kit (Macherey-Nagel), and size selection was carried out to obtain approximately 300 bp of DNA fragments using SPRIselect Reagent (Beckmann Coulter). RT-qPCR was performed using specific primers described in Supplemental Table 7. Libraries were made using KAPA Hyper Prep kit (Roche). Briefly, 30 ng from each immunoprecipitated or input DNA was end-repaired, phosphorylated, A-tailed, and ligated to adaptors. Ligated products were size-selected with 0.8× SPRI beads to obtain 250–350 bp of DNA. After purification, an 8-cycle PCR amplification reaction was performed. PCR product was cleaned using 1× SPRI beads. Sequencing and post-processing of the raw data were performed at the Genomics Resources Core facility of Weill Cornell Medicine.

### ChIP sequencing analysis.

ChIP-Seq data were aligned to human hg19 reference genome using Bowtie v0.12.9 with default parameters (74). Peak calling was performed by macs14 v1.4.2 with default parameters (75). BigWig files of ChIP-Seq data track and analysis of read density in peak regions were generated using deepTools v3.1.3 (76). Read density of specific genomic regions was displayed using Integrative Genomics Viewer v2.4.19 (77). Pathway enrichment analysis was performed by enrichGO function from clusterProfiler v3.2.14. Promoters were defined as the regions encompassing 2 kb in both directions of the transcription start site of University of California Santa Cruz Genomics Institute (UCSC genes).

### Analysis of cytotoxic killing of MCL cells by FOXO1 inhibitors in vitro and in vivo.

Compound 10 (cpd10) was previously published (50, 51). For in vitro treatment, cpd10 was reconstituted in DMSO as 0.8 mM stock and treated for 6 days for cytotoxic responses.

### Data availability.

The data that support the findings of this study are available within the article and its Supplemental Tables. RNA-Seq and ChIP-Seq data can be found in the NCBI’s Gene Expression Omnibus database (GEO GSE182689).

### Statistics.

We determined experimental sample sizes based on preliminary data. For numerical variables, all results are expressed as mean ± SEM. Normal distribution of the sample sets was determined before application of 2-tailed Student’s *t* test or 1-way ANOVA for group comparisons. A 2-sample Student’s *t* test was used to compare the differences between 2 groups, and a 1-way ANOVA was used to assess the differences among multiple groups followed by a post hoc 2-sample Student’s *t* test between groups with a Bonferroni’s procedure to adjust the *P* values for multiple comparisons. Kaplan-Meier method was used to estimate the probability of survival, and the log-rank test was used to compare the overall survival difference between groups. All statistical tests were 2-sided, and the differences were considered significant when *P* was less than 0.05. GraphPad Prism software was used for all statistical analyses.

### Study approval.

All studies involving the use of laboratory mice were approved by the Weill Cornell Medicine Institutional Animal Care and Usage Committee (IACUC protocol 2018-0039).

## Author contributions

IH, HZ, and JP conceived and designed the study. IH, HP, EI, and JP developed methodology. IH, JYJ, MJK, YW, and JP acquired data. IH, JYJ, JY, ZC, KVB, RAB, HP, EI, CZ, YW, MJK, HZ, and JP analyzed and interpreted data (e.g., statistical analysis, biostatistics, computational analysis). IH, JYJ, HP, EI, LA, JY, GI, OE, HZ, and JP wrote the manuscript. RAB, LA, MDL, XH, SCK, LCC, YL, SB, HVL, HP, LM, OE, GI, HZ, and JP provided administrative, technical, or material support (e.g., reporting or organizing data, constructing databases). SB selected the study compound. OE, LM, HZ, and JP supervised the study.

## Supplementary Material

Supplemental data

Supplemental table 1

Supplemental table 2

Supplemental table 3

Supplemental table 4

Supplemental table 5

Supplemental table 6

Supplemental table 7

## Version 1 10/25/2022

In-Press Preview

## Version 2 12/15/2022

Electronic publication

## References

[1] Hill HA (2020). Genetic mutations and features of mantle cell lymphoma: a systematic review and meta-analysis. Blood Adv.

[2] Khouri IF (1998). Hyper-CVAD and high-dose methotrexate/cytarabine followed by stem-cell transplantation: an active regimen for aggressive mantle-cell lymphoma. J Clin Oncol.

[3] Ghielmini M, Zucca E (2009). How I treat mantle cell lymphoma. Blood.

[4] Wang ML (2013). Targeting BTK with ibrutinib in relapsed or refractory mantle-cell lymphoma. N Engl J Med.

[5] Maddocks K, Blum KA (2015). Treatment strategies in mantle cell lymphoma. Cancer Treat Res.

[6] Rule S (2019). Ibrutinib for the treatment of relapsed/refractory mantle cell lymphoma: extended 3.5-year follow up from a pooled analysis. Haematologica.

[7] Wang M (2019). Durable response with single-agent acalabrutinib in patients with relapsed or refractory mantle cell lymphoma. Leukemia.

[8] Song Y (2020). Treatment of patients with relapsed or refractory mantle-cell lymphoma with zanubrutinib, a selective inhibitor of Bruton’s tyrosine kinase. Clin Cancer Res.

[9] Wang M (2020). KTE-X19 CAR T-cell therapy in relapsed or refractory mantle-cell lymphoma. N Engl J Med.

[10] Goy A (2009). Bortezomib in patients with relapsed or refractory mantle cell lymphoma: updated time-to-event analyses of the multicenter phase 2 PINNACLE study. Ann Oncol.

[11] Goy A (2013). Single-agent lenalidomide in patients with mantle-cell lymphoma who relapsed or progressed after or were refractory to bortezomib: phase II MCL-001 (EMERGE) study. J Clin Oncol.

[12] Eyre TA (2021). Therapeutic options for relapsed/refractory mantle cell lymphoma. Blood.

[13] Lee TI, Young RA (2013). Transcriptional regulation and its misregulation in disease. Cell.

[14] Garraway LA, Sellers WR (2006). Lineage dependency and lineage-survival oncogenes in human cancer. Nat Rev Cancer.

[15] Goss PE (2011). Exemestane for breast-cancer prevention in postmenopausal women. New Engl J Med.

[16] Mehta RS (2012). Combination anastrozole and fulvestrant in metastatic breast cancer. N Engl J Med.

[17] de Bono JS (2011). Abiraterone and increased survival in metastatic prostate cancer. N Engl J Med.

[18] Ryan CJ (2013). Abiraterone in metastatic prostate cancer without previous chemotherapy. N Engl J Med.

[19] Zhang Y (2011). FoxO family members in cancer. Cancer Biol Ther.

[20] Paik JH (2007). FoxOs are lineage-restricted redundant tumor suppressors and regulate endothelial cell homeostasis. Cell.

[21] Gan B (2010). FoxOs enforce a progression checkpoint to constrain mTORC1-activated renal tumorigenesis. Cancer Cell.

[22] Yang Y (2017). Loss of FOXO1 cooperates with TMPRSS2-ERG overexpression to promote prostate tumorigenesis and cell invasion. Cancer Res.

[23] Dengler HS (2008). Distinct functions for the transcription factor Foxo1 at various stages of B cell differentiation. Nat Immunol.

[24] Amin RH, Schlissel MS (2008). Foxo1 directly regulates the transcription of recombination-activating genes during B cell development. Nat Immunol.

[25] Herzog S (2008). SLP-65 regulates immunoglobulin light chain gene recombination through the PI(3)K-PKB-Foxo pathway. Nat Immunol.

[26] Yusuf I (2008). KLF4 is a FOXO target gene that suppresses B cell proliferation. Int Immunol.

[27] Dominguez-Sola D (2015). The FOXO1 transcription factor instructs the germinal center dark zone program. Immunity.

[28] Sander S (2015). PI3 kinase and FOXO1 transcription factor activity differentially control B cells in the germinal center light and dark zones. Immunity.

[29] Schmitz R (2012). Burkitt lymphoma pathogenesis and therapeutic targets from structural and functional genomics. Nature.

[30] Trinh DL (2013). Analysis of FOXO1 mutations in diffuse large B-cell lymphoma. Blood.

[31] Bradner JE (2017). Transcriptional addiction in cancer. Cell.

[32] Henley MJ, Koehler AN (2021). Advances in targeting ‘undruggable’ transcription factors with small molecules. Nat Rev Drug Discov.

[33] Lambert SA (2018). The human transcription factors. Cell.

[34] Lu B (2018). A transcription factor addiction in leukemia imposed by the MLL promoter sequence. Cancer Cell.

[35] Tsherniak A (2017). Defining a cancer dependency map. Cell.

[36] Beekman R (2018). SOX11, a key oncogenic factor in mantle cell lymphoma. Curr Opin Hematol.

[37] Balaji S (2018). NF-κB signaling and its relevance to the treatment of mantle cell lymphoma. J Hematol Oncol.

[38] Zhao X (2021). Transcriptional programming drives Ibrutinib-resistance evolution in mantle cell lymphoma. Cell Rep.

[39] Murre C (2018). ‘Big bang’ of B-cell development revealed. Genes Dev.

[40] Ferreirós-Vidal I (2019). Feedforward regulation of Myc coordinates lineage-specific with housekeeping gene expression during B cell progenitor cell differentiation. PLoS Biol.

[41] Chen D (2016). The expression pattern of the pre-B cell receptor components correlates with cellular stage and clinical outcome in acute lymphoblastic leukemia. PLoS One.

[42] Zaret KS, Carroll JS (2011). Pioneer transcription factors: establishing competence for gene expression. Genes Dev.

[43] Hatta M, Cirillo LA (2007). Chromatin opening and stable perturbation of core histone:DNA contacts by FoxO1. J Biol Chem.

[44] Psenakova K (2019). Forkhead domains of FOXO transcription factors differ in both overall conformation and dynamics. Cells.

[45] Shi J (2015). Discovery of cancer drug targets by CRISPR-Cas9 screening of protein domains. Nat Biotechnol.

[46] Tang ED (1999). Negative regulation of the forkhead transcription factor FKHR by Akt. J Biol Chem.

[47] Wang F (2018). Tight regulation of FOXO1 is essential for maintenance of B-cell precursor acute lymphoblastic leukemia. Blood.

[48] Gehringer F (2019). FOXO1 confers maintenance of the dark zone proliferation and survival program and can be pharmacologically targeted in Burkitt lymphoma. Cancers (Basel).

[49] Pyrzynska B (2018). FOXO1 promotes resistance of non-Hodgkin lymphomas to anti-CD20-based therapy. Oncoimmunology.

[50] Lee YK (2021). FOXO1 inhibition synergizes with FGF21 to normalize glucose control in diabetic mice. Mol Metab.

[51] Langlet F (2017). Selective inhibition of FOXO1 activator/repressor balance modulates hepatic glucose handling. Cell.

[52] Zhao X (2015). CCMCL1: a new model of aggressive mantle cell lymphoma. Blood.

[53] Mansson R (2012). Positive intergenic feedback circuitry, involving EBF1 and FOXO1, orchestrates B-cell fate. Proc Natl Acad Sci U S A.

[54] Welinder E (2011). The transcription factors E2A and HEB act in concert to induce the expression of FOXO1 in the common lymphoid progenitor. Proc Natl Acad Sci U S A.

[55] Morin RD (2016). Genetic landscapes of relapsed and refractory diffuse large B-cell lymphomas. Clin Cancer Res.

[56] Morin RD (2011). Frequent mutation of histone-modifying genes in non-Hodgkin lymphoma. Nature.

[57] Pasqualucci L (2014). Genetics of follicular lymphoma transformation. Cell Rep.

[58] Zhou P (2019). Sporadic and endemic Burkitt lymphoma have frequent *FOXO1* mutations but distinct hotspots in the AKT recognition motif. Blood Adv.

[59] Kabrani E (2018). Nuclear FOXO1 promotes lymphomagenesis in germinal center B cells. Blood.

[60] Roberto MP (2021). Mutations in the transcription factor FOXO1 mimic positive selection signals to promote germinal center B cell expansion and lymphomagenesis. Immunity.

[61] van der Heide LP (2005). FoxO6 transcriptional activity is regulated by Thr26 and Ser184, independent of nucleo-cytoplasmic shuttling. Biochem J.

[62] Martins R (2016). Long live FOXO: unraveling the role of FOXO proteins in aging and longevity. Aging Cell.

[63] Kochenderfer JN (2017). Long-duration complete remissions of diffuse large B cell lymphoma after anti-CD19 chimeric antigen receptor T cell therapy. Mol Ther.

[64] Chiron D (2013). Induction of prolonged early G1 arrest by CDK4/CDK6 inhibition reprograms lymphoma cells for durable PI3Kδ inhibition through PIK3IP1. Cell Cycle.

[65] Sanjana NE (2014). Improved vectors and genome-wide libraries for CRISPR screening. Nat Methods.

[66] Hwang I (2021). Cellular stress signaling activates type-I IFN response through FOXO3-regulated lamin posttranslational modification. Nat Commun.

[67] Rubio K (2019). Inactivation of nuclear histone deacetylases by EP300 disrupts the MiCEE complex in idiopathic pulmonary fibrosis. Nat Commun.

[68] Martin M (2011). Cutadapt removes adapter sequences from high-throughput sequencing reads. EMBnet J.

[69] Patro R (2017). Salmon provides fast and bias-aware quantification of transcript expression. Nat Methods.

[70] Robinson MD, Oshlack A (2010). A scaling normalization method for differential expression analysis of RNA-seq data. Genome Biol.

[72] Liberzon A (2015). The molecular signatures database (MSigDB) hallmark gene set collection. Cell Syst.

[73] Hwang I (2020). CIC is a critical regulator of neuronal differentiation. JCI Insight.

[74] Langmead B (2009). Ultrafast and memory-efficient alignment of short DNA sequences to the human genome. Genome Biol.

[75] Zhang Y (2008). Model-based analysis of ChIP-Seq (MACS). Genome Biol.

[76] Ramírez F (2016). deepTools2: a next generation web server for deep-sequencing data analysis. Nucleic Acids Res.

[77] Robinson JT (2011). Integrative genomics viewer. Nat Biotechnol.

