# Supplementary material for: A FOXO1-dependent transcription network is a targetable vulnerability of mantle cell lymphomas
Source: J Clin Invest. 2022 Dec 15;132(24):e160767. doi: 10.1172/JCI160767 (PMC9753996; doi:10.1172/JCI160767)
Supplement: Supplemental data [file jci-132-160767-s179.pdf]

Supplemental Figure 1-7

A FOXO1-dependent transcription network is a targetable vulnerability of mantle cell lymphomas

Supplemental Figure 1

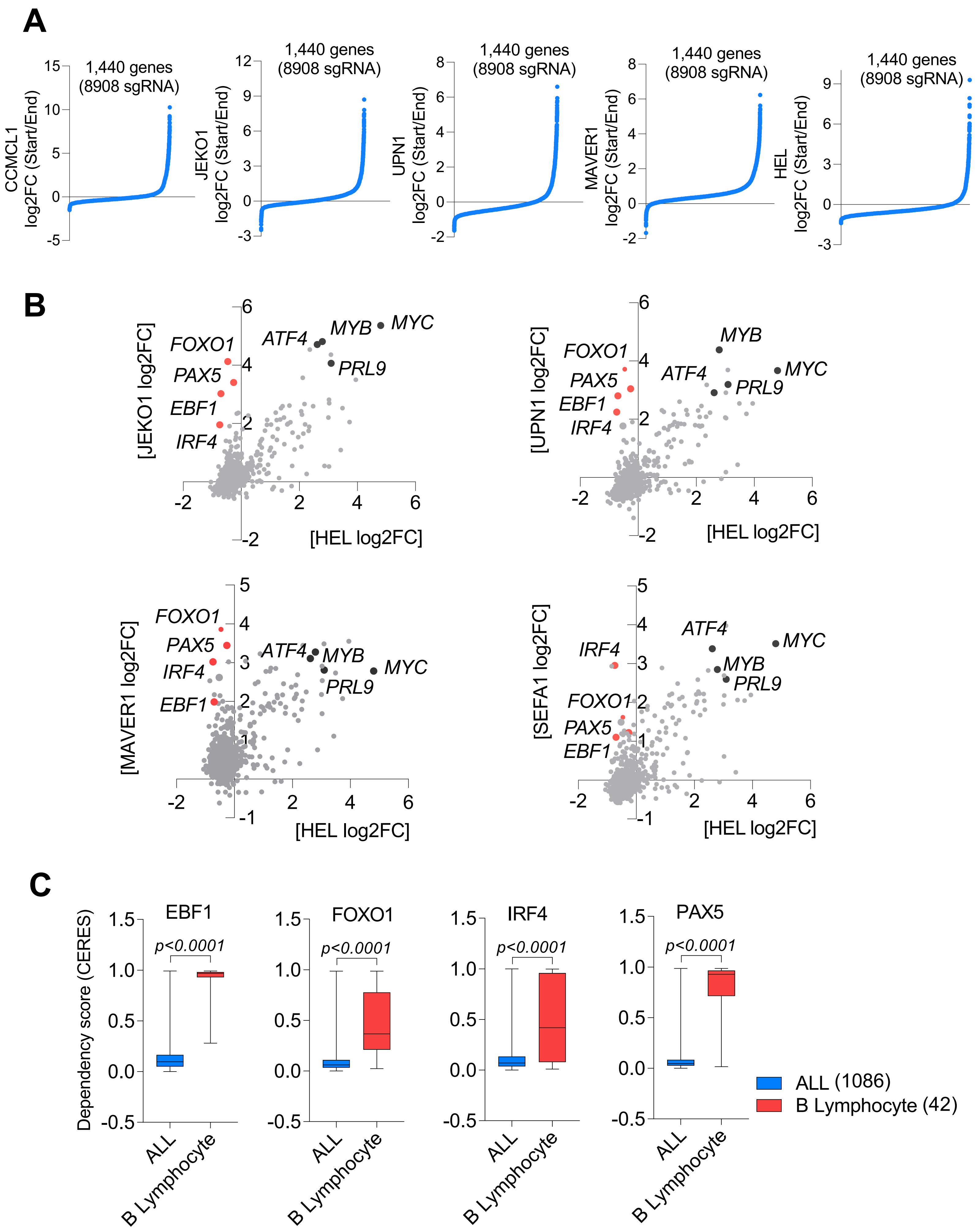

Supplemental Figure 1

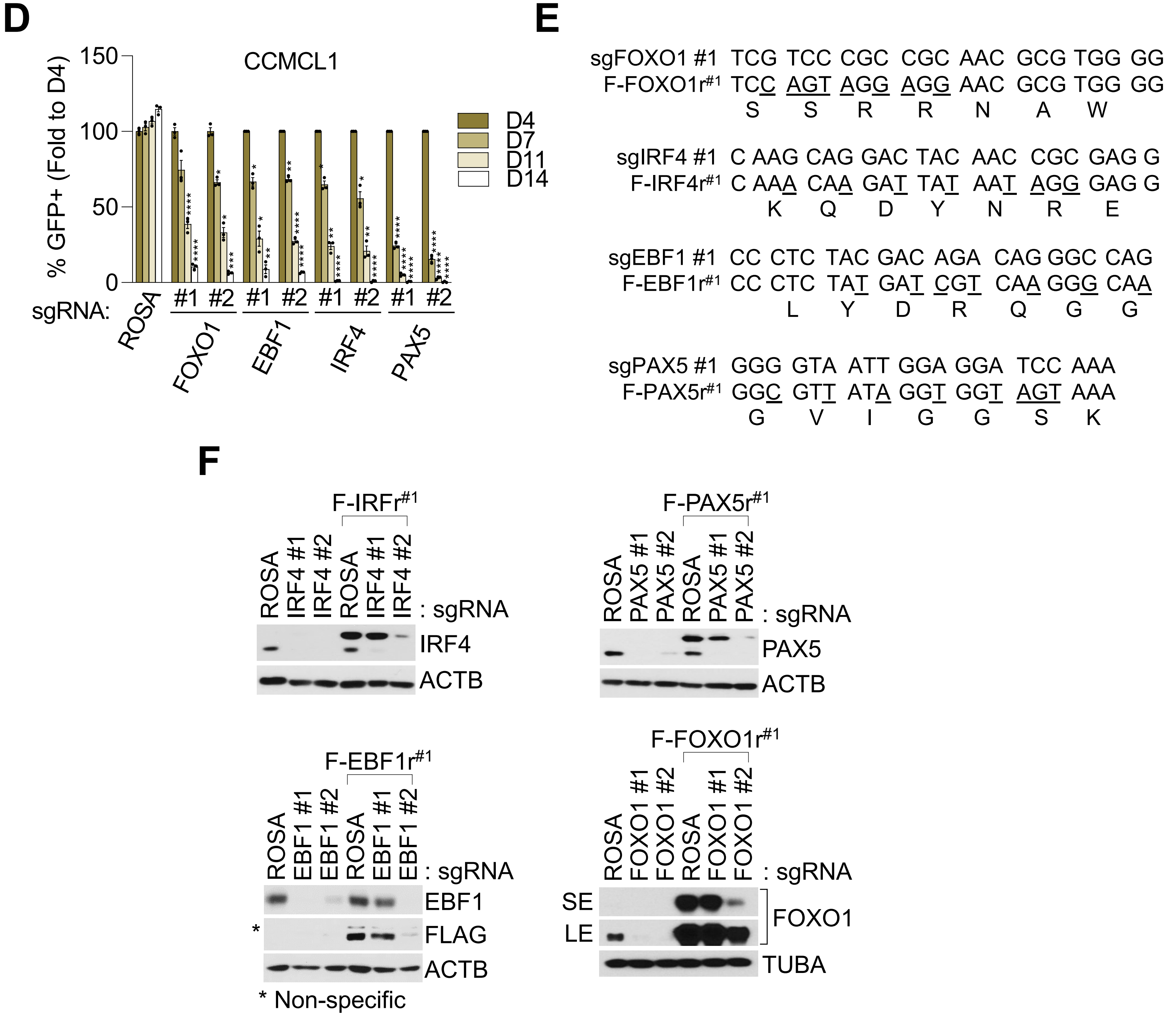

Supplemental Figure 1. CRISPR/Cas9 screening identifies core survival transcription factors of MCL cells. (A) Ranking of the log2 fold-change (log2FC) of sgRNA abundance in CCMCL1, JEKO1, UPN1, MAVER1, and HEL cells (Ratio of start to end point) after 14 population doublings. (B) Scatterplot analysis of TF dependencies in JEKO1, UPN1, MAVER, and SEFA1 (Y axis) comparing to HEL (X axis) by the average sgRNA log2FC of each genes in the pooled CRISPR screen. (C) Dependency scores of four TFs extracted from Project Achilles (22Q2, ref 35). The box plots indicate the distribution of dependency score (CERES, a normalized metric of gene essentiality) of four TFs across all 1,086 cell lines or in the subset of 42 cell lines of B-lymphocyte origin. Data represent mean ± SEM. P value was calculated using a two-tailed unpaired Student's t-test. (D) Competition-based proliferation assays to validate the results from the pooled screen. Experiments were conducted by transduction of Cas9-expressing CCMCL1. Data represent mean ± SEM (n=3). Results are representative of 2 independent experiments. Statistical analysis was performed using 1-way ANOVA with Tukey's multiple-comparison test. ****$p<0.0001$, ***$p<0.0005$, **$p<0.005$, *$p<0.05$. (E) The design of the indicated CRISPR-resistant synonymous mutant. (F) Immunoblot performed at day 3 upon transduction of indicated sgRNAs. SE and LE denote short exposure and long exposure, respectively.

Supplemental Figure 2

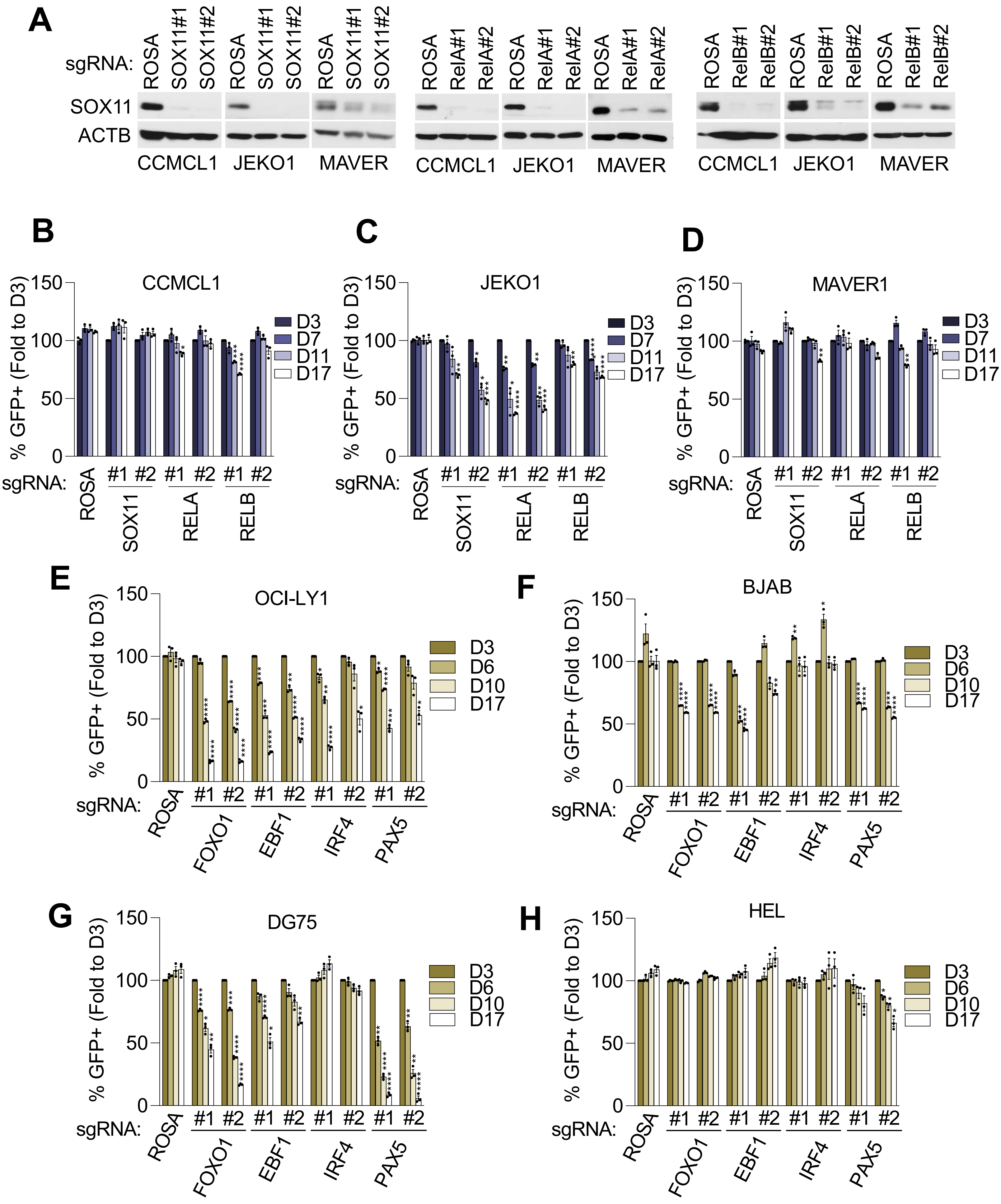

Supplemental Figure 2

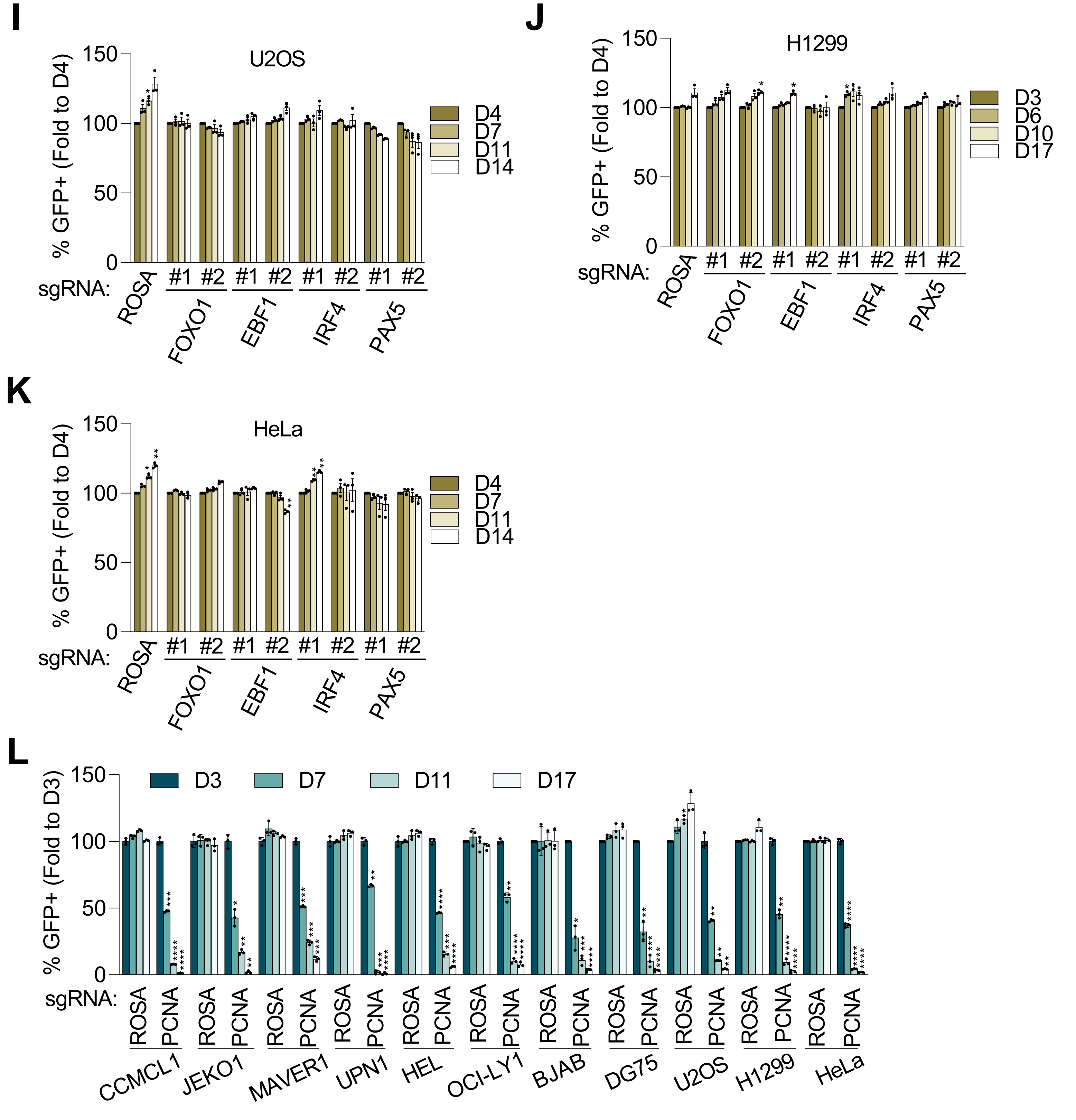

Supplemental Figure 2. IRF4, PAX5, EBF1 and FOXO1 are MCL lineage-survival TFs. (A) Immunoblot performed at day 7 upon transduction of indicated sgRNAs. (B-L) Competition-based proliferation assays to validate the results from the pooled screen. Experiments were conducted by transduction of Cas9-expressing CCMCL1 (B), JEKO1 (C), MAVER (D), OCI-LY1 (E), BJAB (F), DG75 (G), HEL (H), U2OS (I), H1299 (J), and HeLa (K) cells with indicated lentivirus sgRNAs that co-express a GFP reporter. (L) PCNA positive control in all cell lines. Plotted are the GFP% cells (normalized to the day 3 measurement) at the indicated time-points during culturing. In B, C, E, F, G, H, and L, data represent mean ± SEM (n=3). Results are representative of 2 or 3 independent experiments. Statistical analysis was performed using 1-way ANOVA with Turkey's multiple-comparison test. *****p<0.0001, ***p<0.0005, **p<0.005, *p<0.05.*

A

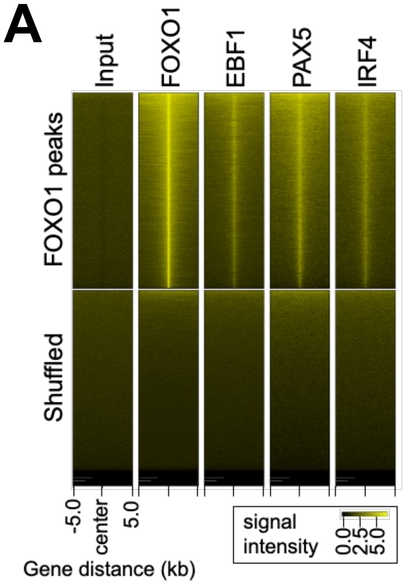

B

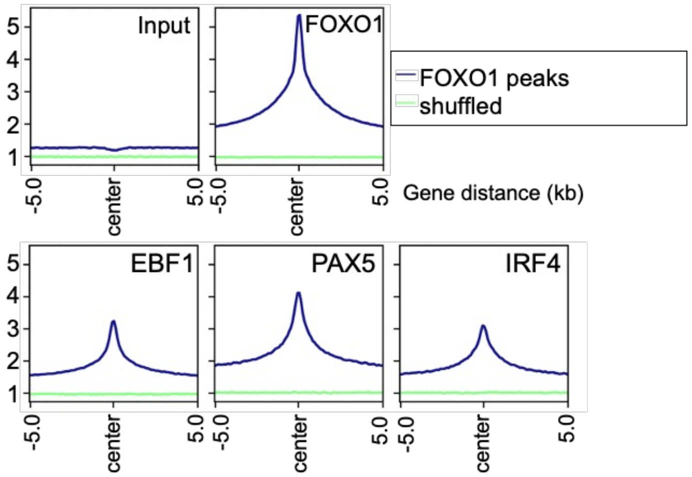

C

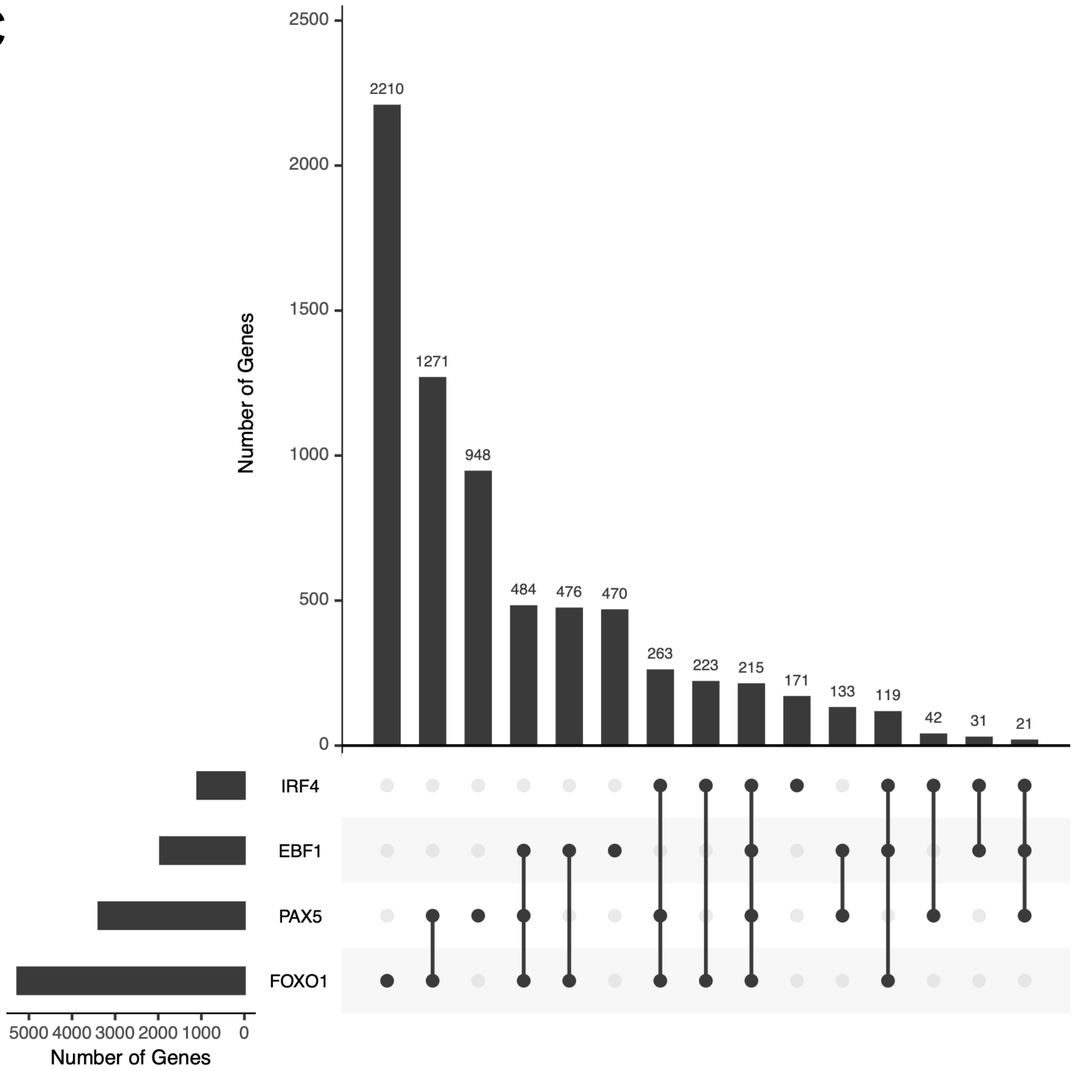

FOXO1	EBF1	PAX5	IRF4	Group	# of genes
100%	24.596%	42.444%	15.586%	FOXO1	5261
66.393%	100%	43.766%	19.805%	EBF1	1949
66.123%	25.259%	100%	16.02%	PAX5	3377
75.576%	35.576%	49.861%	100%	IRF4	1085

Supplemental Figure 3

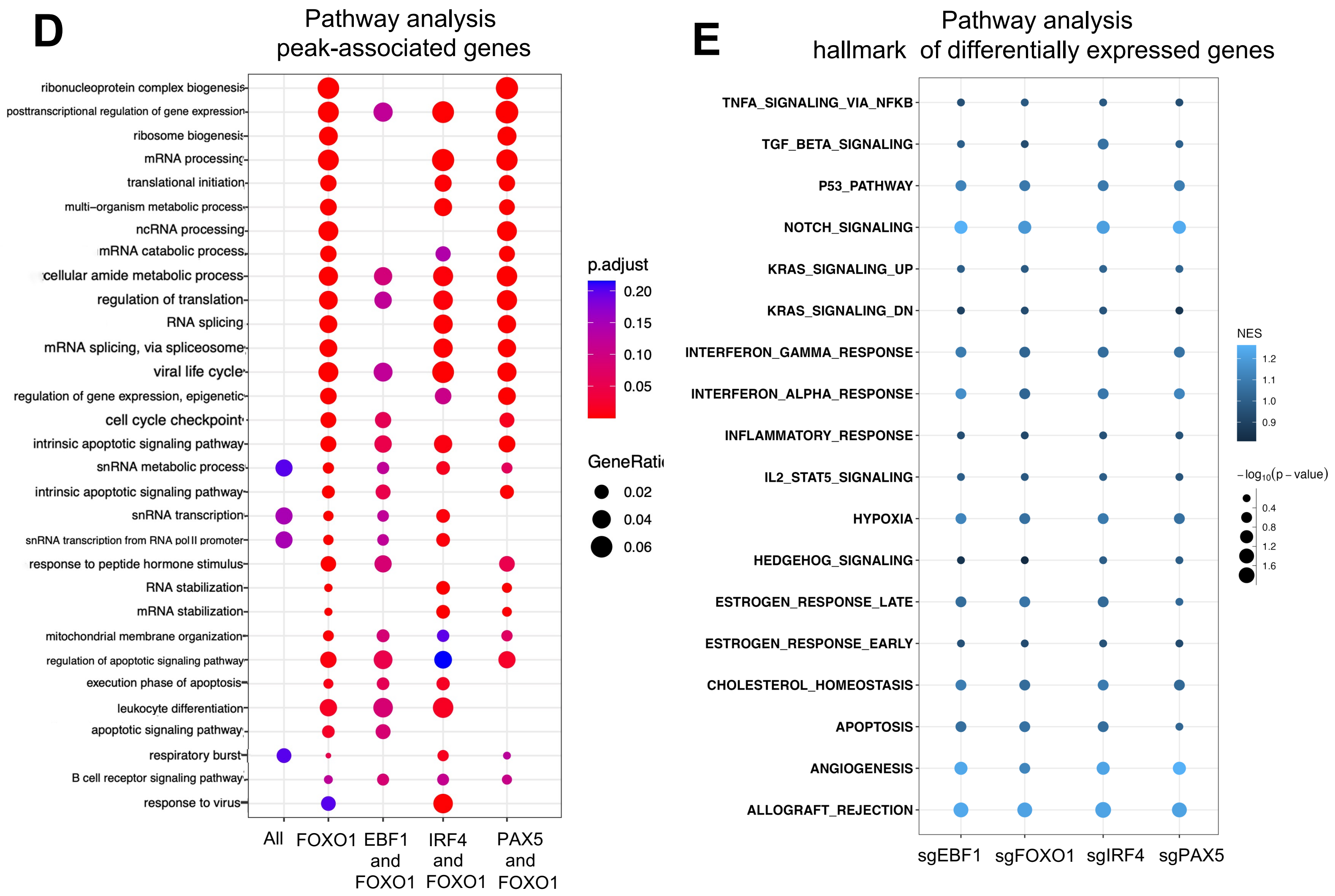

Supplemental Figure 3. Analysis of co-occupancy of 4 TFs and Gene Ontology analysis of RNA-seq data from FOXO1-depleted CCMCL1. (A, B) Validation of ChIP-seq analysis. Peak enrichment was confirmed against shuffled peaks. (C) The UpSet plot depicting the intersection of genes occupied by the four factors. Percent overlap is indicated in the table. (D) Gene Ontology enrichment analysis of peak-associated genes. Gene ratios of pathway annotated genes in peak-associated genes are indicated by the size of the dot, and adjusted p-values are indicated by color according to the key to the right (a hypergeometric test followed by a Benjamini-Hochberg procedure). (E) RNA-seq analysis of CCMCL1 cells expressing specific sgRNA for each TF (96 h) was performed. RNA-seq data were analyzed for enrichment levels of selected gene sets from the MSigDB hallmarks collection. Significance and enrichment score are represented by circle size (-log10 of p-value) and color gradient (normalized enrichment score).

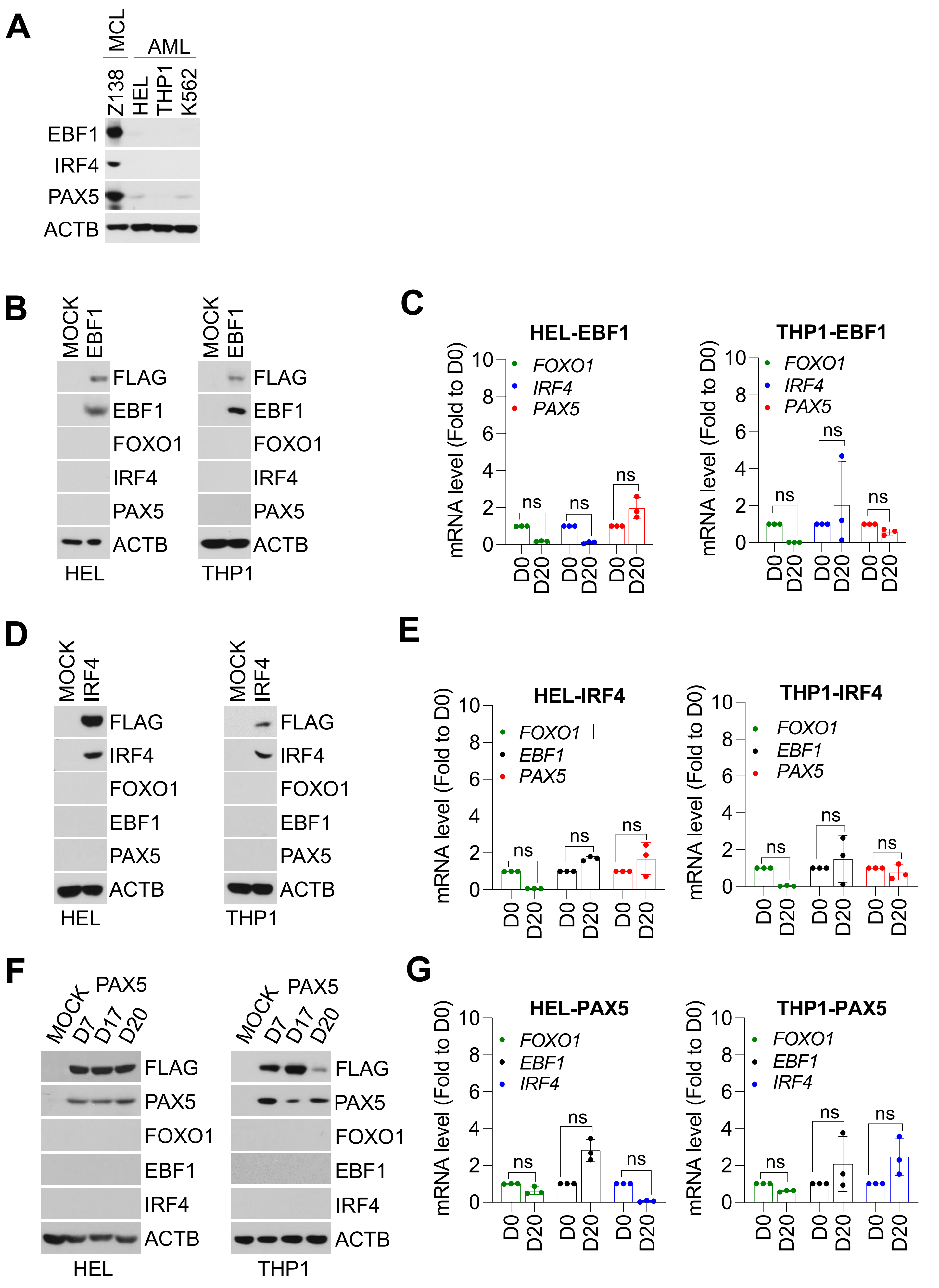
A
MCL
AML
Z138
HEL
THP1
K562
EBF1
IRF4
PAX5
ACTB
B
MOCK
EBF1
FLAG
EBF1
FOXO1
IRF4
PAX5
ACTB
HEL
THP1
C
HEL-EBF1
THP1-EBF1
mRNA level (Fold to D0)
FOXO1
IRF4
PAX5
ns
D0
D20
D
MOCK
IRF4
FLAG
IRF4
FOXO1
EBF1
PAX5
ACTB
HEL
THP1
E
HEL-IRF4
THP1-IRF4
FOXO1
EBF1
PAX5
F
MOCK
PAX5
D7
D17
D20
FLAG
PAX5
FOXO1
EBF1
IRF4
ACTB
HEL
THP1
G
HEL-PAX5
THP1-PAX5
FOXO1
EBF1
IRF4

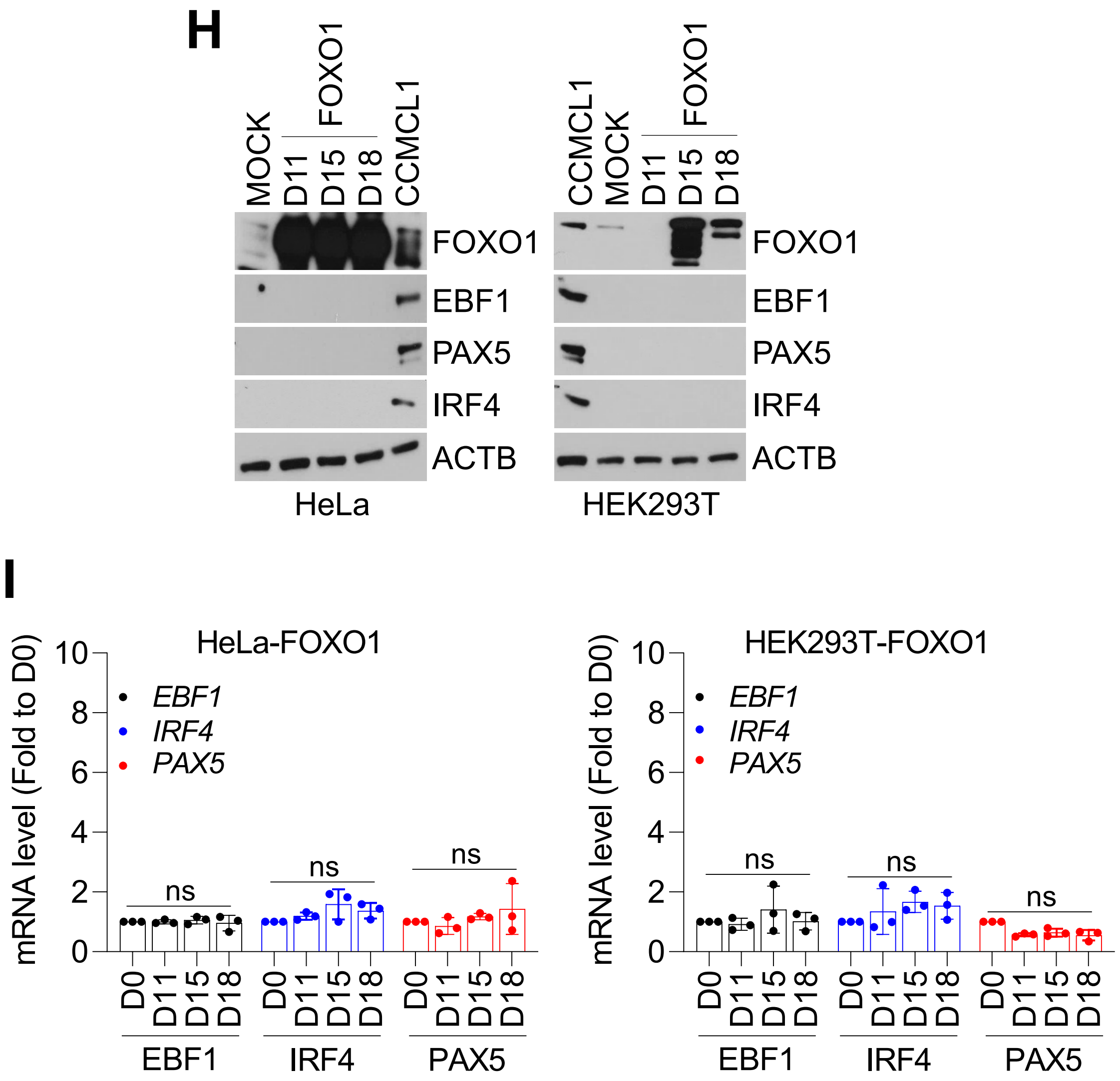

Supplemental Figure 4. Ectopic expression of EBF1, IRF4, or PAX5 does not activate MCL lineage-survival TF program in HEL or THP1 cells. (A) Immunoblot analysis of EBF1, IRF4, and PAX5 expression in the indicated cell lines. (B, C) Immunoblot (B) and RT-qPCR analysis (C) of *FOXO1, IRF4*, and *PAX5* expression in EBF1-transduced HEL or THP1 cells. In C, data represent mean ± SEM (n=3). (D, E) Immunoblot (D) and RT-qPCR analysis (E) of *FOXO1, EBF1*, and *PAX5* expression in IRF4-transduced HEL or THP1 cells. In E, data represent mean ± SEM (n=3). (F, G) Immunoblot (F) and RT-qPCR analysis (G) of *FOXO1, EBF1*, and *IRF4* expression in PAX5-transduced HEL or THP1 cells. In G, data represent mean ± SEM (n=3). (H, I) Immunoblot (H) and RT-qPCR analysis (I) of *EBF1, IRF4*, and PAX5 expression in FOXO1-transduced Hela or HEK293T cells. Lysates and total RNA were prepared at day 20 post-transduction of indicated genes. In I, data represent mean ± SEM (n=3). (C, E, G, I) Results are representative of 3 independent experiments. Statistical analysis in C, E, and G was performed using two-tailed unpaired Student's *t*-test and I using 1-way ANOVA with Tukey's multiple-comparison test. ns, no significant.

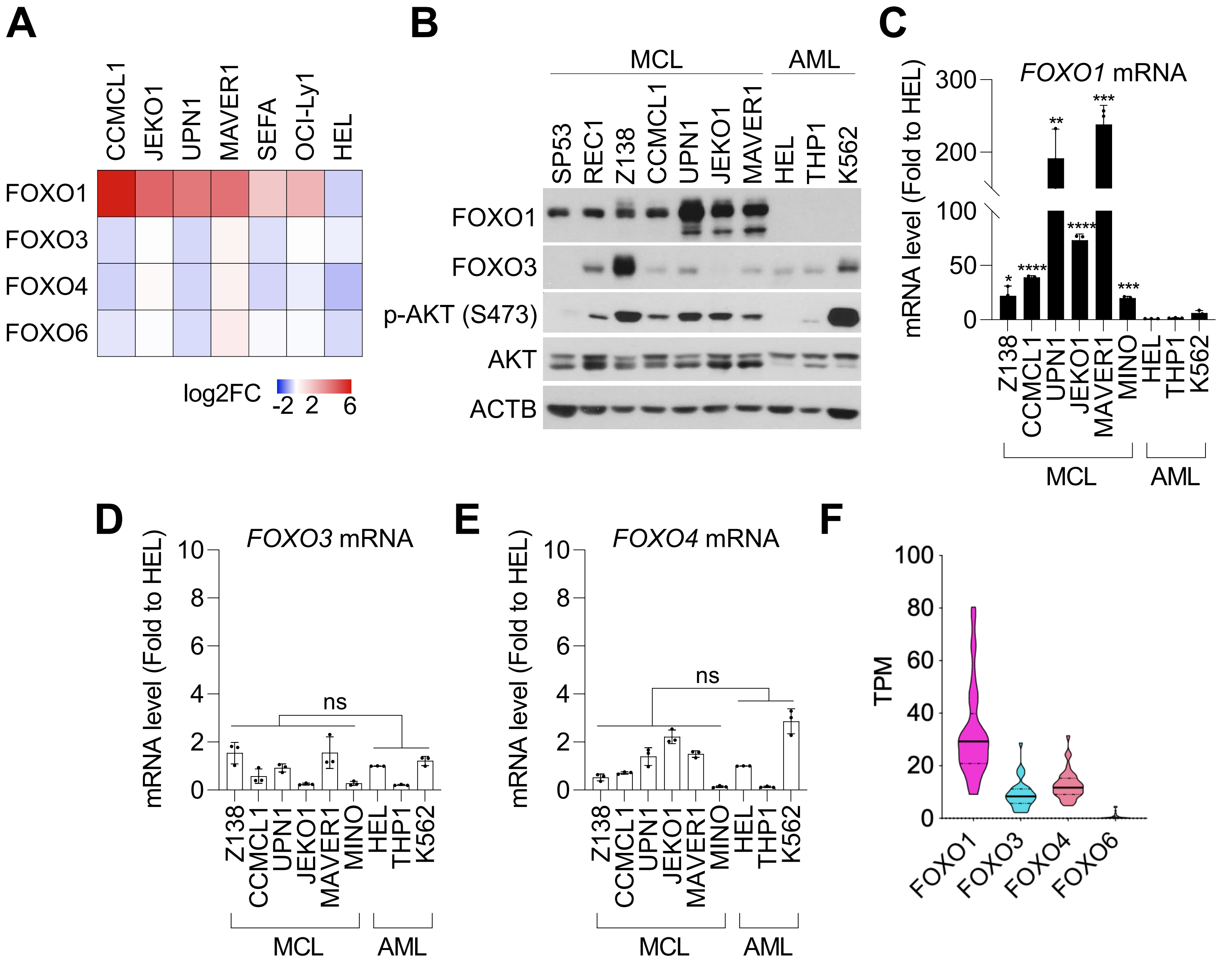

Supplemental Figure 5. FOXO1 but not FOXO isoforms is required for MCL maintenance. (A) Heatmap depicts log2FC of sgRNA abundance of FOXO1, FOX3, FOXO4, and FOXO6 (averaging each independent sgRNA targeting a gene). (B) Immunoblot analysis of MCL and AML cell lines for the indicated gene expression. (c-e) RT-qPCR analysis of FOXO1 (C), FOXO3 (D), and FOXO4 (E) mRNA expression in the indicated MCL or AML cell lines. Data represent mean ± SEM (n=3). Results are representative of 3 independent experiments. Statistical analysis was performed using 1-way ANOVA with Tukey's multiple-comparison test. Each MCL cell line was compared with the mean values of the three AML cell lines.*****p<0.0001, ***p<0.0005, **p<0.005, *p<0.05*. ns, no significant. (F) Violin plots of RNA expression levels in transcripts per million (TPM) of FOXO isoforms in patient MCL cells (n=37). RNA-seq data were reanalyzed from GSE141336 of Zhao et al.

A

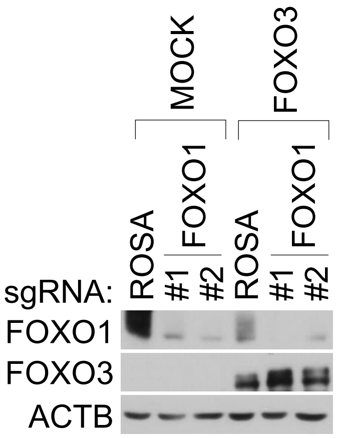

B

```
Score            Expect    Method                          Identities      Positives       Gaps
454 bits(1168)   4e-156    Compositional matrix adjust.    315/707(45%)    416/707(58%)    86/707(12%)

Query  1    MAEAPQV--------VEIDPDFEPLPRPRSCTWPLPRPEFSQSNSATSSPA-PSGSAAAN  51
            MAEAP          VE+DP+FEP  RPRSCTWPL RPE        +SPA PSG  AA+
Sbjct  1    MAEAPASPAPLSPLEVELDPEFEPQSRPRSCTWPLQRPELQ------ASPAKPSGETAAD  54

Query  52   ---PDAAAGLPSASAAAVSADFMSNLSLLEESEDFPQAPGSVAAAVAA-------AAAAA  101
               P+             +   M+                   +A V A       +  A
Sbjct  55   SMIPEEEDDEDDEDGGGRAGSAMAIGGGGGSGTLGSGLLLEDSARVLAPGGQDPGSGPAT  114

Query  102  ATGGLCGDFQGPEAGCLHPAPPQPPPPGPLSQHPPVPPAAAGPLAGQPRKSSSSRRNAWG  161
            A GGL G   G +A      P  PP PG          AA G  +GQPRK SS RRNAWG
Sbjct  115  AAGGLSG---GTQALLQPQQPLPPPQPG----------AAGG--SGQPRKCSS-RRNAWG  158

Query  162  NLSYADLITKAIESSAEKRLTLSQIYEWMVKSVPYFKDKGDSNSSAGWKNSIRHNLSLHS  221
            NLSYADLIT+AIESS +KRLTLSQIYEWMV+ VPYFKDKGDSNSSAGWKNSIRHNLSLHS
Sbjct  159  NLSYADLITRAIESSPDKRLTLSQIYEWMVRCVPYFKDKGDSNSSAGWKNSIRHNLSLHS  218

Query  222  KFIRVQNEGTGKSSWWMLNPEGGKSGKSPRRRAASMDNNSKFAKSRSRAAKKKASLQSGQ  281
            +F+RVQNEGTGKSSWW++NP+GGKSGK+PRRRA SMDN++K+ KSR RAAKKKA+LQ+
Sbjct  219  RFMRVQNEGTGKSSWWIINPDGGKSGKAPRRRAVSMDNSNKYTKSRGRAAKKKAALQTAP  278

Query  282  EGAGDSPGSQFSKWPASPGSHSNDDFDNWSTFRPRTSSNASTISGRLSPIM--TEQDDLG  339
            E A DSP SQ SKWP SP S S+D+ D W+ FR RT+SNAST+SGRLSPIM  TE D++
Sbjct  279  ESADDSP-SQLSKWPGSPTSRSSDELDAWTDFRSRTNSNASTVSGRLSPIMASTELDEVQ  337

Query  340  EGD--VHSMVYPPSAAKMAST-------LPSLSEISNPENM-----ENLLDNL--NLLSS  383
            + D  +  M+Y  SA+   S        LP L++++   N+     ENL+D+L  N+
Sbjct  338  DDDAPLSPMLYSSSASLSPSVSKPCTVELPRLTDMAGTMNLNDGLTENLMDDLLDNITLP  397

Query  384  PTSLTVSTQSSP-GTMMQQTPCYSFAPPNTSLNSPSPNYQKYTYGQSSMSPLPQMPIQTL  442
            P      +Q SP G +MQ++  + +    + L SP+ ++    +G SS++ L Q P+QT+
Sbjct  398  P------SQPSPTGGLMQRSSSFPYTTKGSGLGSPTSSFNSTVFGPSSLNSLRQSPMQTI  451

Query  443  QDNK-SSYGGMSQYNCAPGLLKELLTSDSPPHNDI-MTPVDPGVAQPNSRVLGQNV---M  497
            Q+NK +++  MS Y      L++LLTSDS  H+D+ MT  DP ++Q ++ V  QN    +
Sbjct  452  QENKPATFSSMSHYGNQ--TLQDLLTSDSLSHSDVMMTQSDPLMSQASTAVSAQNSRRNV  509

Query  498  MGPNSVMSTYGSQASHNKMMNPSSHTHPGHAQQTSAVNGRPLPHTVSTMPHTSGMNRLTQ  557
            M  N  M ++ +Q +   ++N  +  H  H  Q +    R L ++VS M  +   + L
Sbjct  510  MLRNDPMMSFAAQPNQGSLVN-QNLLHHQHQTQGALGGSRALSNSVSNMGLSE-SSSLGS  567

Query  558  VKTPVQVPLPHPMQMSALGGYSSVSSCNGYGRMGLLHQEKLPSDLD-GMFIERLDCDMES  616
             K   Q P+   MQ +     S  S  +    + ++  EK PSDLD  MF   L+CDMES
Sbjct  568  AKHQQQSPVSQSMQ-TLSDSLSGSSLYSTSANLPVMGHEKFPSDLDLDMFNGSLECDMES  626

Query  617  IIRNDLMDGDTLDFNFDNVLPNQ--------SFPHSVKTTTHSWVSG  655
            IIR++LMD D LDFNFD+++  Q        +F  + + ++ SWV G
Sbjct  627  IIRSELMDADGLDFNFDSLISTQNVVGLNVGNFTGAKQASSQSWVPG  673
```

C

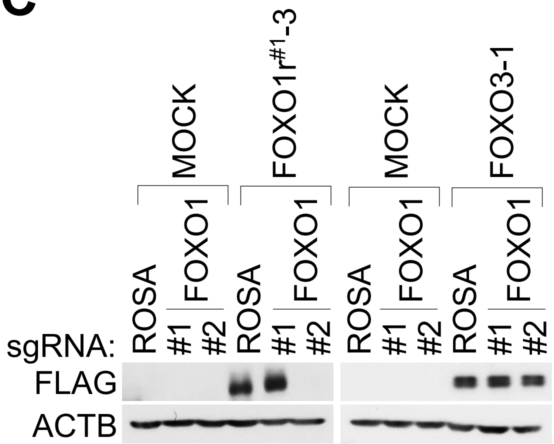

D

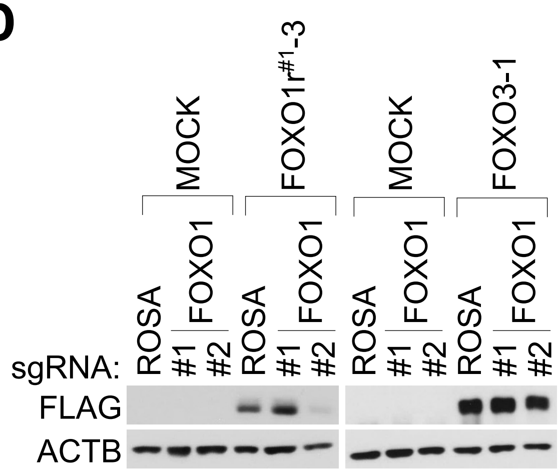

Supplemental Figure 6

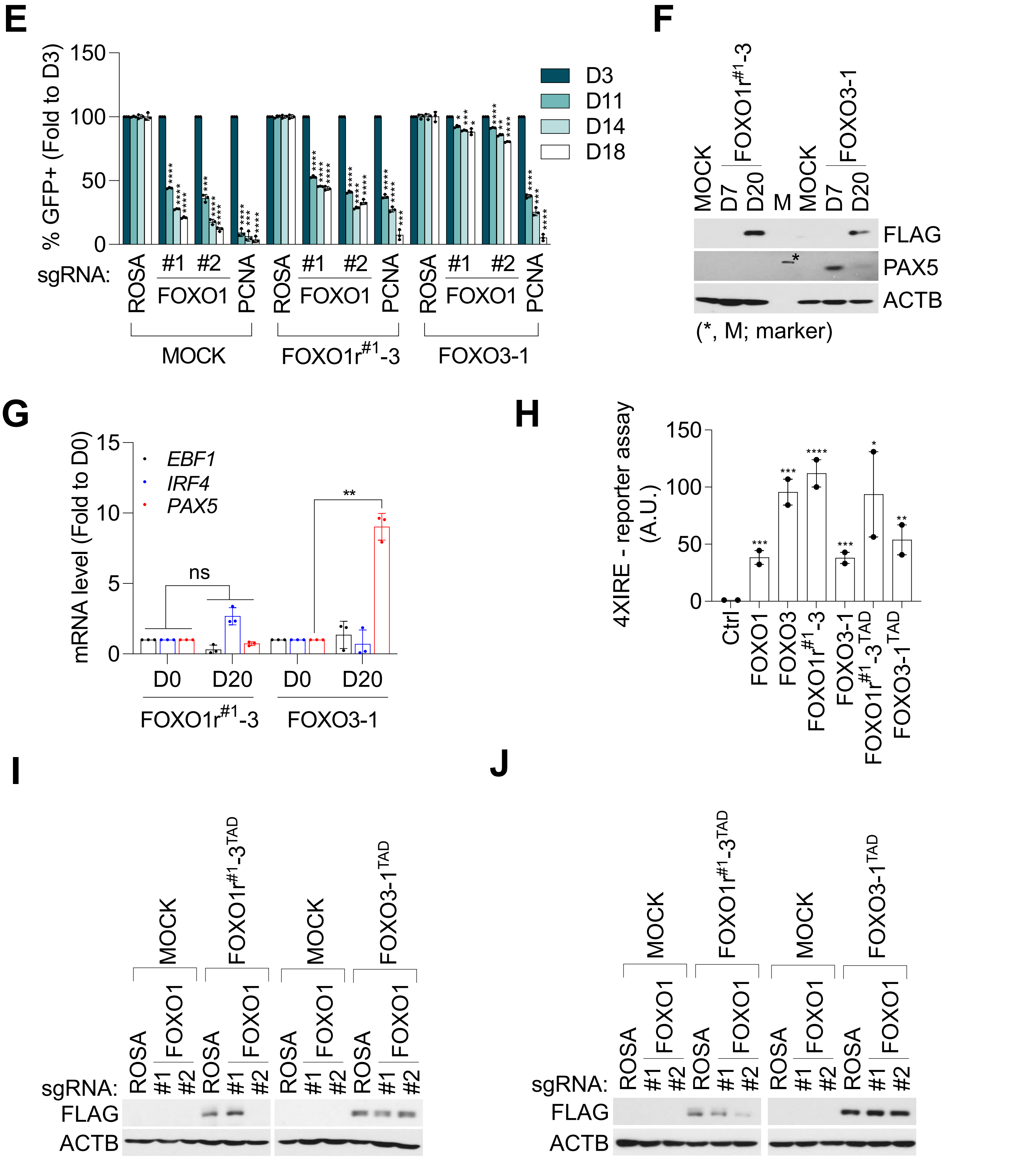

K

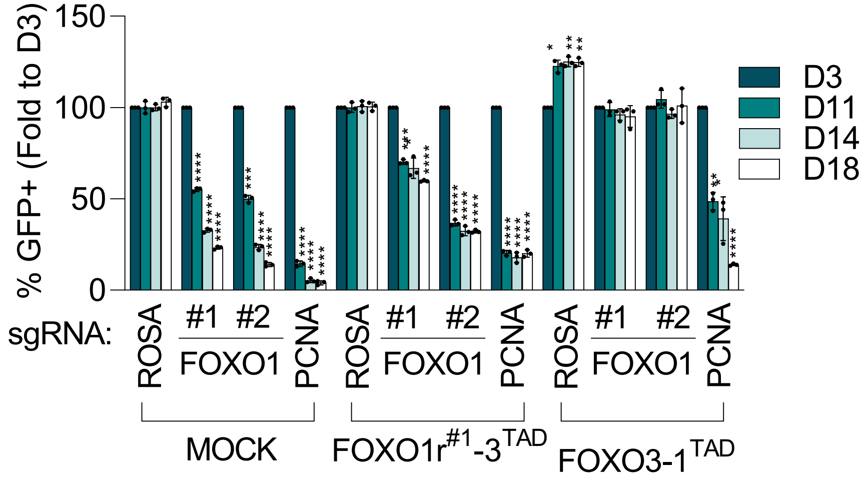

L

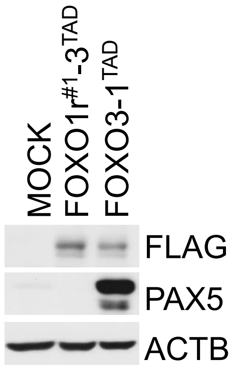

M

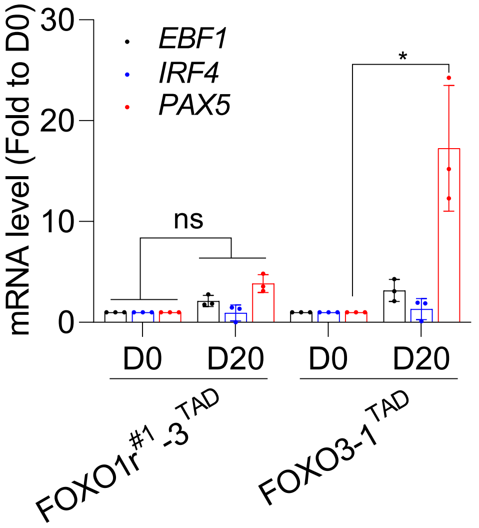

Supplemental Figure 6. TAD motif of FOXO1 is crucial for its MCL supporting activity. (A) Immunoblot analysis of control and FOXO3 expressing CCMCL1 cells with indicated sgRNA. (B) Sequence alignment between FOXO1 and FOXO3 proteins. Blue and pink box indicate DBD and TAD, respectively. (C, D) Immunoblot analysis of control and 3xFlag-tagged FOXO1r$^{\#1}$-3 (left) or FOXO3-1 (right) transduced CCMCL1 (C) and UPN1 (D) cells at day 3 following transduction of indicated sgRNAs. (E) Competition-based proliferation assays in FOXO1r$^{\#1}$-3 (left) or FOXO3-1 (right) transduced UPN1 cells. Data represent mean ± SEM (n=3). (F, G) Immunoblot (F) and RT-qPCR (G) analysis of *EBF1, IRF4* and *PAX5* induction in FOXO1r-3- (left) or FOXO3-1-transduced (right) HEL cells. Cell lysates and total RNA were prepared at day 7 post-infection of lentivirus encoding indicated variants. In G, data represent mean ± SEM (n=3). Results are representative of 3 independent experiments. (H) FOXO1-driven 4XIRE-luciferase reporter analysis of HEK293T cells co-transfected with indicated FOXO variants. Data represent mean ± SEM (n=3). (I, J) Immunoblot analysis of control and 3xFlag-tagged FOXO1r$^{\#1}$-3^{TAD} (left) or FOXO3-1^{TAD} (right) transduced CCMCL1 (I) and UPN1 (J) cells at day 3 following transduction of indicated sgRNAs. (K) Competition-based proliferation assays in FOXO1r$^{\#1}$-3^{TAD} (left) or FOXO3-1^{TAD} (right) transduced UPN1 cells. Data represent mean ± SEM (n=3). (L, M) Immunoblot (L) and RT-qPCR (M) analysis of *EBF1, IRF4* and *PAX5* induction in FOXO1r$^{\#1}$-3^{TAD}- (left) or FOXO3-1^{TAD}-transduced (right) HEL cells. Cell lysates and total RNA were prepared at day 7 post-infection of lentivirus encoding indicated variants. In M, data represent mean ± SEM (n=3). Results are representative of 3 independent experiments. (E, G, H, K, M) Statistical analysis in E, K was performing using 1-way ANOVA with Tukey's multiple-comparison test and G, H, M using two-tailed unpaired Student's *t*-test. ****$p<0.0001$, ***$p<0.0005$, **$p<0.005$, *$p<0.05$. ns, no significant.

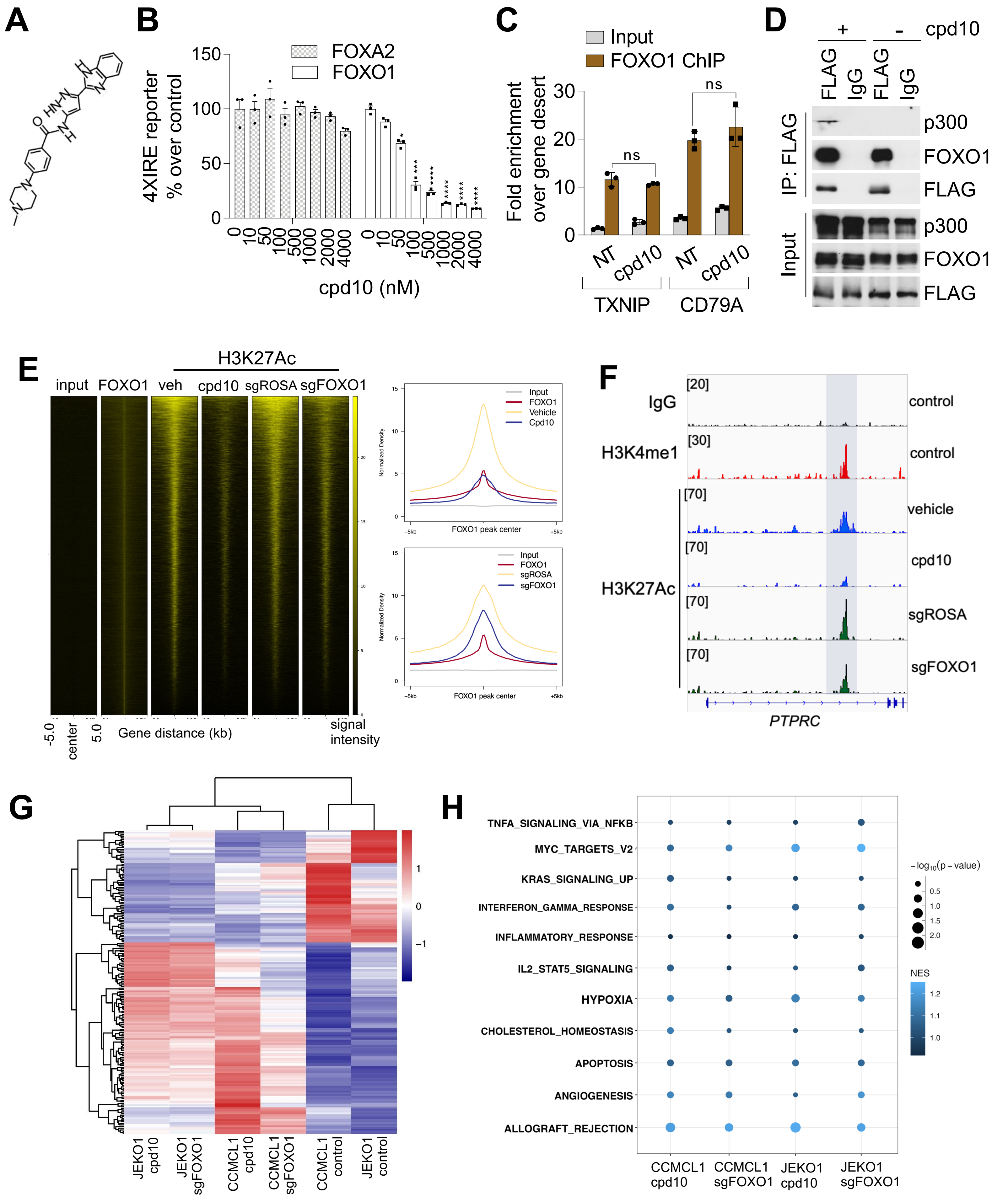
A
B
FOXA2
FOXO1
4XIRE reporter % over control
cpd10 (nM)
C
Input
FOXO1 ChIP
Fold enrichment over gene desert
ns
NT
cpd10
TXNIP
CD79A
D
+
-
cpd10
FLAG
IgG
IP: FLAG
Input
p300
FOXO1
FLAG
E
H3K27Ac
input
FOXO1
veh
cpd10
sgROSA
sgFOXO1
-5.0
center
5.0
Gene distance (kb)
signal intensity
Normalized Density
FOXO1 peak center
Input
FOXO1
Vehicle
Cpd10
sgROSA
sgFOXO1
F
IgG
H3K4me1
H3K27Ac
control
vehicle
cpd10
sgROSA
sgFOXO1
PTPRC
G
JEKO1 cpd10
JEKO1 sgFOXO1
CCMCL1 cpd10
CCMCL1 sgFOXO1
CCMCL1 control
JEKO1 control
H
TNFA_SIGNALING_VIA_NFKB
MYC_TARGETS_V2
KRAS_SIGNALING_UP
INTERFERON_GAMMA_RESPONSE
INFLAMMATORY_RESPONSE
IL2_STAT5_SIGNALING
HYPOXIA
CHOLESTEROL_HOMEOSTASIS
APOPTOSIS
ANGIOGENESIS
ALLOGRAFT_REJECTION
−log10(p−value)
NES
CCMCL1 cpd10
CCMCL1 sgFOXO1
JEKO1 cpd10
JEKO1 sgFOXO1

Figure

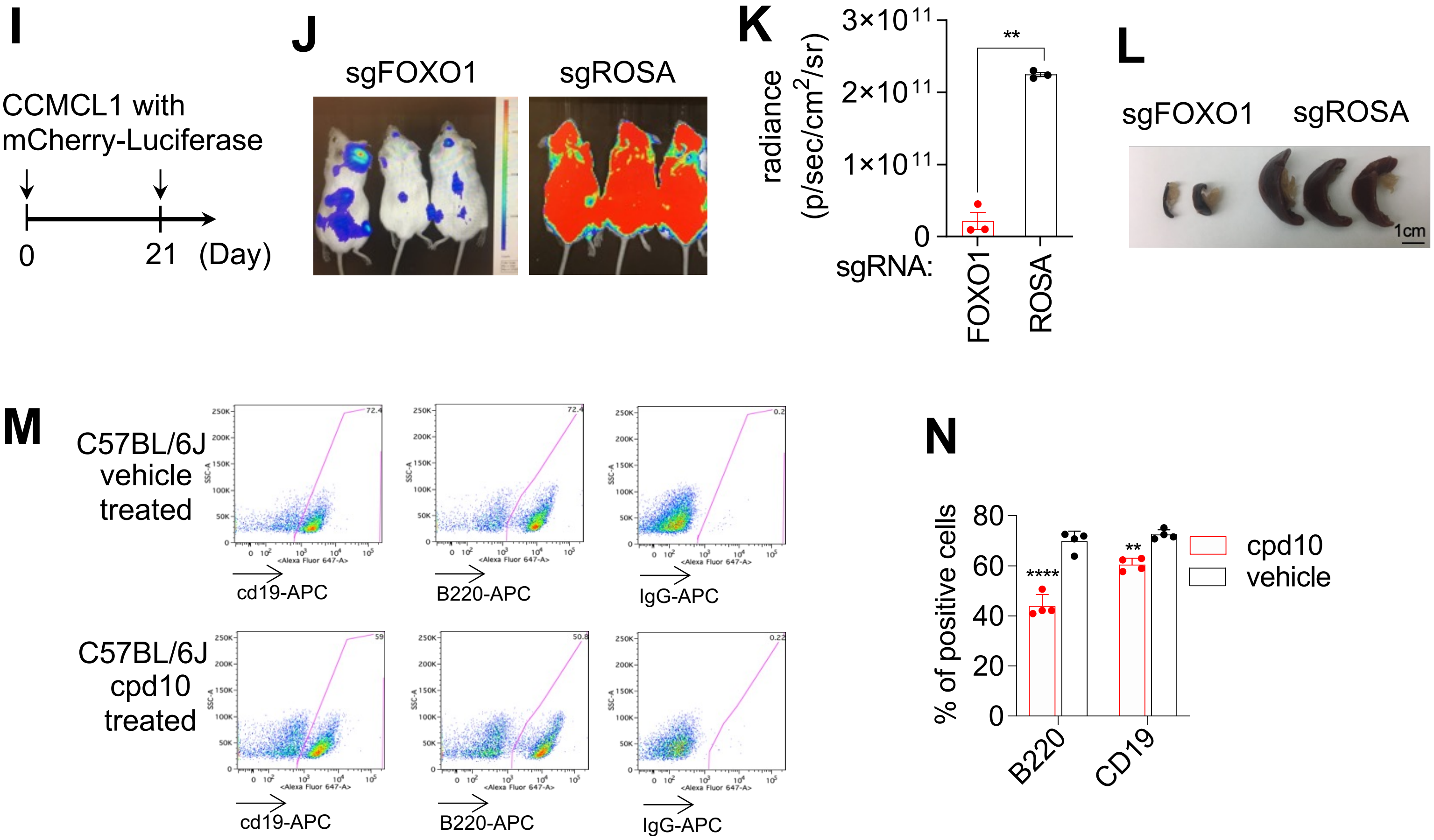

Supplemental Figure 7. Depletion of FOXO1 suppresses MCL tumorigenesis in vivo. (A) cpd10 (B) 4XIRE-luciferase reporter assay in FOXO1 or FOXA2-transfected HEK293T cells at 48 h post cpd10 treatment. Mean ± SEM (n=3). (C) FOXO1 ChIP-qPCR of CCMCL1 cells with or without 2 μM cpd10 treatment for 4 h. Mean ± SEM (n=3). (D) Co-immunoprecipitation of FOXO1 with p300. Flag-FOXO1-expressing CCMCL1 cells were treated with 2 μM cpd10 or not for 24 h. (E) Left, heatmap of signals from ChIP-seq from CCMCL1 cells extending 5 kb in each direction from the FOXO1 peak center. Right, a histogram view of the data. Cells were treated for 48 h with 2 μM cpd10 or FOXO1 depletion by sgFOXO1. (F) ChIP-seq tracks for *PTPRC* locus. (G) Heatmap for the expression of 184 differentially regulated (FC>1.5), FOXO1 peak-associated genes in JEKO1 or CCMCL1 treated with cpd10 or sgFOXO1 for 96 h. The expression values were scaled in the row direction. (H) Enrichment levels of selected gene sets from the hallmarks collection from MSigDB. Significance and enrichment score are represented by circle size (-log10 of p-value) and color gradient (normalized enrichment score). (I) Schematic of experimental design. (J) BLI of mice inoculated with sgRNA-transduced CCMCL1 cells at day 21 post-transplantation. (K) Quantification of BLI as in (J). Mean ± SEM (n=3). (L) A representative image of spleens from mice inoculated with sgROSA- or sgFOXO1-transduced CCMCL1 cells at day 21 post-transplantation. (M, N) B cell content analysis in 10-day vehicle or cpd10 (100 mg/kg/day i.p.) treated C57BL/6J mouse spleen. Representative flowcytometry analysis (M) or mean± s.d. (n=4) values of vehicle- or cpd10-treated mice (N). Statistical analysis in B was performed by 1-way ANOVA with Tukey's multiple-comparison test and C, K, N by two-tailed unpaired Student's *t*-test. *****p<0.0001, ***p<0.0005, **p<0.005, *p<0.05*. ns, no significant.
