# Supplementary material for: A FOXO1-dependent transcription network is a targetable vulnerability of mantle cell lymphomas
Source: J Clin Invest. 2022 Dec 15;132(24):e160767. doi: 10.1172/JCI160767 (PMC9753996; doi:10.1172/JCI160767)
Supplement: Supplemental table 6 [file jci-132-160767-s185.pdf]

Supplemental Table 6. PK properties of cpd 10

Cpd 10 mouse PK		
IV (1 mg/kg)	T1/2	1.23 h
	AUCinf	1.86 μM*h
	Vss	1.71 L/kg
	CL	22.7 mL/kg*min
IP (50 mg/kg)	Tmax	0.58 h
	Cmax	2.18 μM
	AUCinf	17.61 μM*h

PK: pharmacokinetics. IV: intravenous. IP: Intraperitoneal. CL: clearance. Vss: steady state volume of distribution.
