# Supplementary material for: A FOXO1-dependent transcription network is a targetable vulnerability of mantle cell lymphomas
Source: J Clin Invest. 2022 Dec 15;132(24):e160767. doi: 10.1172/JCI160767 (PMC9753996; doi:10.1172/JCI160767)
Supplement: Supplemental table 7 [file jci-132-160767-s186.pdf]

Supplemental Table 7.

REAGENT	SOURCE	IDENTIFIER
**Antibodies**		
FOXO1	Custom made rabbit polyclonal (GL Biochem)	Epitope: Cys-RNDLMDGDTLDFNFDNVLPNQSFPHSVK
FOXO1	Cell Signaling Technology	Cat# 2880
IRF4	Cell Signaling Technology	Cat# 4964
PAX5	Cell Signaling Technology	Cat# 12709
EBF1	Millipore Sigma	Cat# ABE1294
Beta actin (ACTB)	Millipore Sigma	Cat# A2228
FOXO3	Cell Signaling Technology	Cat# 12829
V5	ThermoFisher Scientific	Cat# 377500
FLAG	Millipore Sigma	Cat# F1804
Akt (pan)	Cell Signaling Technology	Cat# 4685
p-Akt (S473)	Cell Signaling Technology	Cat# 4060
Alpha Tubulin (TUBA)	Abcam	Cat# ab7291
EP300	Cell signaling Technology	Cat#54062
Alexa 488 anti-mouse	ThermoFisher Scientific	Cat# A21202
Alexa 488 anti-rabbit	ThermoFisher Scientific	Cat# A21206
Goat anti-mouse IgG HRP	ThermoFisher Scientific	Cat# 31430
Goat anti-rabbit IgG HRP	Cell Signaling Technology	Cat# 7074
**Bacterial Strain**		
Stable Competent E. coli	New England BioLabs	Cat# C3040H
**Chemicals and biologicals**		

Dulbecco's Modification of Eagle's Medium (DMEM)	Corning	Cat# 10-013-CV
RPMI 1640	ThermoFisher scientific	Cat# 11875-093
GlutaMAX	ThermoFisher scientific	Cat# 35050061
Penicillin/Streptomycin	ThermoFisher scientific	Cat# 10378016
USDA approved fetal bovine serum (FBS)	Avantor Seradigm	Cat# 89510-186
Typsin EDTA	Corning	Cat# 25-053-CI
Puromycin	InvivoGen	Cat# anti-pr-1
Blasticidin	InvivoGen	Cat# anti-bl-1
Geneticin® Selective Antibiotic (G418 Sulfate)	Thermo Fisher Scientific	Cat# 11811031
Polyethyleneimine (PEI)	Polysciences Inc	Cat# 23966-1
Opti-MEM™ I Reduced Serum Medium	Thermo Fisher Scientific	Cat# 31985070
SPRIselect Reagent	Beckman Coulter	Cat# B23317
CloneAmp™ HiFi PCR Premix	Clontech	Cat# 639298
Gibson Assembly Mix	New England BioLabs Inc.	Cat# E2611L
Q5®High-Fidelity 2X mix	New England BioLabs Inc.	Cat# M0492L
TaKaRa Ex Taq® DNA Polymerase	TAKARA	Cat# RR001C
NucleoSpin Gel and PCR clean-up DNA extraction kit	Macherey-Nagel (MN)	Cat# 740609.25
NucleoBond Xtra Maxi kit	Macherey-Nagel (MN)	Cat# 740414.50
NucleoBond Xtra Midi kit	Macherey-Nagel (MN)	Cat# 740410.50

NucleoSpin Plasmid EasyPure	Macherey-Nagel (MN)	Cat# 74027.25
NucleoSpin RNA kit	Macherey-Nagel (MN)	Cat# 740955.25
PowerUP SYBR Green Mix	ThermoFisher Scientific	Cat# A25778
RevertAid RT Reverse Transcription kit	ThermoFisher Scientific	Cat# K1691
Dual-Luciferase® Reporter Assay System	Promega	Cat# E1910
Proteinase K	Macherey-Nagel (MN)	Cat# 740506
16% paraformaldehyde	ThermoFisher Scientific	Cat# 28908
Dynabeads® Protein G	ThermoFisher Scientific	Cat# 1009D
EGS	ThermoFisher Scientific	Cat# 21565
Ultrapure™ Phenol	ThermoFisher Scientific	Cat# 15509037
2-Mercaptoethanol	Sigma-Aldrich	Cat# M6250
Bovine Serum Albumin	Sigma-Aldrich	Cat# A3059
Pierce BCA protein assay kit	ThermoFisher Scientific	Cat# 23225
PVDF transfer membrane	ThermoFisher Scientific	Cat# 88518
Nitrocellulose Blotting Membrane	GE Healthcare Life science	Cat# 10600003
SuperSignal West Pico Chemiluminescent substrate	ThermoFisher Scientific	Cat# 34080
Anti-DYKDDDDK Magnetic Agarose	ThermoFisher Scientific	Cat#A36797
Protein A Agarose Resin	Gold Biotechnology	Cat#P-400-S
**Experimental Models: Cell Lines**		
CCMCL1	Chen-Kiang Laboratory	CVCL_RU10

JEKO1	ATCC	CRL-3006
UPN1	Expasy	CVCL_A795
MAVER-1	ATCC	CRL-3008
Z-138	ATCC	CRL-3001
MINO	ATCC	CRL-3000
K562	ATCC	CRL-3343
REC1	ATCC	CRL-3004
LCL9001	Coriell Institute	LCL-9001
SP53	Expasy	CVCL_C122
SEFA	OSU, Baiocchi Laboratory	
OCI-Ly1	Expasy	CVCL_1879
THP1	ATCC	TIB-202-AP1-LUC2
HEL	ATCC	TIB-180
BJAB	ATCC	JHU-32
DG75	ATCC	CRL-2625
H1299	ATCC	CRL-5803
HeLa	ATCC	CCL-2
U2OS	ATCC	HTB-96
HEK293T	ATCC	CRL-11268
**Oligonucleotides**		
sgFOXO1#1	TCGTCCCGCCGCAACGCGTG	
sgFOXO1#2	GTCGCAGATCTACGAGTGGA	
sgEBF1#1	GCTGGCCCTGTCTGTCGTAG	

sgEBF1#2	CCTGGCCCTCTACGACAGAC
sgIRF4#1	CAAGCAGGACTACAACCGCG
sgIRF4#2	GGTACTTGCCGCTGTCGATC
sgMYC#1	GAGGCGAACACACAACGTCT
sgMYC#2	ACAACGTCTTGGAGCGCCAG
sgPAX5#1	TTGGATCCTCCAATTACCCC
sgPAX5#2	TCCCGGATGTAGTCCGCCAG
sgSOX11#1	CGATGAACGCGTTCATGGTA
sgSOX11#2	CGAGAAGATCCCGTTCATCC
sgRELA#1	GTGACAGTGCGGGACCCATC
sgRELA#2	AAGCGCATGCCCCGCTGCTT
sgRELB#1	AAGCGCATGCCGCGCTGCTT
sgRELB#2	ACTGCACCGACGGCATCTGC
sgROSA	GAAGATGGGCGGGAGTCTTC
sgPCNA	GGACTCGTCCCACGTCTCTT
hFOXO1 -For	CTACGAGTGGATGGTCAAGAGC
hFOXO1-Rev	CCAGTTCCTTCATTCTGCACACG
hFOXO3-For	TCTACGAGTGGATGGTGCGTTG
hFOXO3-Rev	CTCTTGCCAGTTCCCTCATTCTG
hFOXO4 -For	ACGAGTGGATGGTCCGTACTGT
hFOXO4-Rev	CCTTGATGAACTTGCTGTGCAGG
hCXCR4-For	CTCCTCTTTGTCATCACGCTTCC
hCXCR4-Rev	GGATGAGGACACTGCTGTAGAG

hCD79B-For	AAGGCTGGCATGGAGGAAGATC	
hCD79B-Rev	GTGCTCACCTACAGACCACTTC	
hEBF1-For	ATGTCTCCGAGGCATCACAAGC	
hEBF1-Rev	TCATGCTCGTGGTGACGGAGTT	
hIRF4-For	GAACGAGGAGAAGAGCATCTTCC	
hIRF4-Rev	CGATGCCTTCTCGGAACTTTCC	
hPAX5-For	CTTGCTCATCAAGGTGTCAGGC	
hPAX5-Rev	TGGCGACCTTTGGTTTGGATCC	
hACTB-For	CACCATTGGCAATGAGCGGTTC	
hACTB-Rev	AGGTCTTTGCGGATGTCCACGT	
hPTPRC-For	CTTCAGTGGTCCCATTGTGGTG	
hPTPRC-Rev	CCACTTTGTTCTCGGCTTCCAG	
chTXNIP-For	CAGCGATCTCACTGATTGGT	
chTXNIP-Rev	GGAGGCGGAAACGTCTCTAT	
chCD79A-For	TCTCTCCACTCACAGCCTGA	
chCD79A-Rev	CAAACCCCACCCTACTTCCT	
**Recombinant DNA**		
pMD2.G	Trono Lab	Addgene #12259
psPAX2	Trono Lab	Addgene #12260
LentiCrisprV2	[59]	Addgene #52961
LRG2.1 (U6-sgRNA-GFP)	[60]	Addgene #108098
pLu-3XFLAG-FOXO1r		Homemade
pLu-3XFLAG-EBF1r		Homemade

pLu-3XFLAG-IRF4r		Homemade
pLu-3XFLAG-PAX5r		Homemade
pLu-3XFLAG-FOXO1r H215R		Homemade
pLu-3xFLAG-EBF1		Homemade
pLX304-EBF1-V5		Homemade
pLu-3xFLAG-IRF4		Homemade
pLu-3xFLAG-PAX5		Homemade
pLu-3xF-FOXO3		Homemade
pLu-3xF-FOXO1r-3(N-C)_298		Homemade
pLu-3xF-FOXO3-1(N-C)_296		Homemade
pLu-3xF-FOXO1r-3(N-C)_TAD		Homemade
pLu-3xF-FOXO3-1(N-C)_TAD		Homemade
pRL-CMV		Promega
pGL4.26-4xIRE-luc2		Accili Lab
**Software, Algorithms and Instruments**		
GraphPad Prism		Version 9
ImageJ	[61]	https://imagej.nih.gov/ij/
7500 fast software	ThermoFisher Scientific	Version2.3
7500 Fast Real-time PCR system		Cat# 4351106
EVOS FL Cell Auto Imaging System	ThermoFisher Scientific	Cat# AMAFD1000
LSR II Flow Cytometer	BD Biosciences	Cat# 642221
M220 Focused-ultrasonicator	Covaris	

IVIS Spectrum Image Analysis	PerkinElmer Inc	Cat# 124262
Living Image®	PerkinElmer Inc	Version 4.7.3
